# The index ‘Treatment Duration Control’ for enabling randomized controlled trials with variation in duration of treatment of chronic pain patients

**DOI:** 10.1186/1471-2288-13-123

**Published:** 2013-10-11

**Authors:** Hilbert W van der Glas, Robert J van Grootel

**Affiliations:** 1 Department of Otorhinolaryngology and Head & Neck Surgery, University Medical Center Utrecht, G05.129, P.O. Box 85500, 3508 GA, Utrecht, The Netherlands

**Keywords:** Randomized trial methodology, Decision rules, Routine outcome monitoring, Treatment duration, Chronic pain, Temporomandibular disorders, Quality of life, EQ-5D

## Abstract

**Background:**

Treatment duration varies with the type of therapy and a patient’s recovery speed. Including such a variation in randomized controlled trials (RCTs) enables comparison of the actual therapeutic potential of different therapies in clinical care. An index, Treatment Duration Control (TDC) of outcome scores was developed to help decide when to end treatment and also to determine treatment outcome by a blinded assessor. In contrast to traditional Routine Outcome Monitoring which considers raw score changes, TDC uses relative change.

**Methods:**

Our theory shows that if a patient with the largest baseline scores in a sample requires a relative decrease by treatment factor *T* to reach a zone of low score values (functional status), any patient with smaller baselines will attain functional status with *T*. Furthermore, the end score values are proportional to the baseline. These characteristics concur with findings from the literature that a patient’s assessment of ‘much improved’ following treatment (related to attaining functional status) is associated with a particular relative decrease in pain intensity yielding a final pain intensity that is proportional to the baseline. Regarding the TDC-procedure: those patient’s scores that were related to pronounced signs and symptoms, were selected for adaptive testing (reference scores). A Contrast-value was determined for each reference score between its reference level and a subsequent level, and averaging all Contrast-values yielded TDC. A cut-off point related to factor *T* for attaining functional status, was the TDC-criterion to end a patient’s treatment as being successful. The use of TDC has been illustrated in RCT data from 118 chronic pain patients with myogenous Temporomandibular Disorders, and the TDC-criterion was validated.

**Results:**

The TDC-criterion of successful/unsuccessful treatment approximated the cut-off separating two patient subgroups in a bimodal post-treatment distribution of TDC-values. Pain intensity decreased to residual levels and Health-Related Quality of Life (HRQoL) increased to normal levels, following successful treatment according to TDC. The post-treatment TDC-values were independent from the baseline values of pain intensity or HRQoL, and thus independent from the patient’s baseline severity of myogenous Temporomandibular Disorders.

**Conclusions:**

TDC enables RCTs that have a variable therapy- and patient-specific duration.

## Background

Temporomandibular Disorders (TMD) are characterized by chronic facial pain and restricted jaw movements. Therapies have been evaluated in randomized controlled trials (RCTs) after a constant period of treatment of 6 to 10 weeks in TMD studies [1-4]. However, the duration of treatment varies in clinical care as it depends on the type of therapy as well as on a patient’s speed of recovery. When therapies on TMD differ in mean duration, a constant period of evaluation might influence an assessment of success rate and efficacy of therapies. A short period will favour short therapies whereas a long period might be disadvantageous by including post-treatment changes in success rate. Thus allowing variation of treatment duration complies with clinical care and enables an unbiased comparison of the therapeutic potential of different therapies in RCTs. Such RCTs are especially important for non-life threatening disorders like TMD, which enable a stepped-care approach.

The raw change in scores of measuring instruments has traditionally been considered rather than relative change to determine the effect of treatment. Two conditions characterize the raw change that is clinically relevant [5]. First, a statistically Reliable Change (RC) should exceed the change caused by chance fluctuations, denoted as the Smallest Detectable Difference [6,7] (SDD; thus RC > SDD). Second, a patient should consider the change beneficial [8]. A patient’s functional status corresponds with a sufficiently low severity level of signs and symptoms. A reliable change in scores by which at least the upper limit of a functional status is attained yields a criterion for a successful treatment [9]. Such a criterion is likely concomitant with beneficial change. In clinical care, a clinician will emphasize the attainment of a functional status for ending a treatment as being successful [10].

Routine Outcome Monitoring (ROM) has been introduced in psychiatric care to assess a patient’s progress during treatment [11]. ROMs use questionnaires as measuring instruments and consider raw changes and normative levels of total score values to define Reliable Change (RC) and functional status. ROM helps a clinician to decide when to end a treatment as being ‘successful’, i.e. if both RC has occurred and an upper limit of functional status has been passed.

In order to characterize therapy outcome, three types of variables are of interest which can be obtained by a ROM-procedure but not entirely by a traditional RCT with a constant duration of treatment. The first variable is the time and number of visits needed to come to the occasion at which a patient’s treatment is ended and the decision occurs on a successful/unsuccessful treatment. The second variable is success rate which is based on the dichotomous outcome of successful/unsuccessful treatment of various patients from a therapy group. The third variable is therapy efficacy which is based on the magnitude of an outcome variable of a measuring instrument averaged across patients. A combination of data on treatment duration and number of visits needed, success rate and therapy efficacy are of interest for a costs-effectiveness-analysis.

ROM data allows the determination of differences in success rate and efficacy between therapies in a more natural context than that of a traditional randomized controlled trial. Like in clinical care, therapy duration need not to be fixed and the selection of patients might be less stringent in terms of co-morbidity. ROM data have been used in an RCT in which the efficacy of brief therapy for mood and anxiety disorders was compared to that of usual treatment of longer duration [12]. Furthermore, ROM has enabled the comparison of the outcome of treatment for mild to moderate depression between RCTs and usual clinical care [13].

A patient with a high level of signs and symptoms must show a larger improvement in raw score level to pass the Upper Limit of Functional Status (ULFS), than a patient with lower levels. A large improvement in score level is likely concomitant with a patient’s perception of a large effect of treatment. In contrast, a patient whose score level at baseline is located just above ULFS (at a distance of the Smallest Detectable Difference, SDD), and whose score level passes just below ULFS with Reliable Change (RC > SDD), likely perceives a smaller effect of treatment. This perceived smaller effect may be non satisfactory for a patient when the patient’s expectation of treatment effect is large. The expectation of, for example, patients with facial pain or fibromyalgia regarding treatment of their symptoms is large indeed, i.e. on average 60% for domains pain, fatigue, distress or interference with daily activities [14]. A possible discrepancy between a favourable ROM outcome and a patient’s expectation of treatment effect might increase the risk on relapse for patients with smaller baselines in particular.

The present paper describes a procedure of controlling treatment duration in which relative change rather than raw change in score levels is used. Following a score reduction by a constant factor, the Upper Limit of Functional Status (ULFS) is then passed. Furthermore, the end levels of patients with a small baseline level will be closer to the zero level hence more remote from ULFS than with a traditional ROM. Because of lower end levels, patients with small baselines will perceive more treatment effect with the procedure using relative change than with a traditional ROM.

Findings on the relationship between decrease in pain intensity following treatment and the patients’ assessment of treatment effect [15], strongly suggest that using relative change for describing treatment progress is relevant for chronic pain patients. This relationship has been examined for patients from 10 chronic pain studies in which a randomized administration of pregabin versus a placebo was applied. The patient groups differed in disease, trial duration and demographic characteristics. Patients were stratified by categories of assessment of treatment effect, and the mean change in pain intensity was determined for each category yielding the relationship between change in pain intensity and assessment of treatment effect. When patients were stratified by pain intensity at baseline, the relationship between raw change in pain intensity and assessed treatment effect diverged for the various levels of baseline pain. In contrast, similar relationships occurred when relative (percentage) change in pain intensity was considered (cf Figures six and seven in reference [15]). Thus the degree of improvement by treatment is similarly assessed by chronic pain patients, regardless of their baseline of pain intensity and other differences in their backgrounds and study conditions, when a particular relative decrease in pain intensity has occurred.

If a successful treatment is related to a patient’s assessment of, for example, ‘much improved’ or better, this assessment will be related to attaining a particular relative decrease in pain intensity. Suppose that, like in Temporomandibular Disorders, the Upper Limit of Functional Status (ULFS) of a disease or disorder is characterized by a low level of signs and symptoms of pain and impairment that might occasionally occur in healthy subjects. Then, the amount of relative decrease in pain intensity which is related to the assessment of ‘much improved’ is also likely related to the relative decrease required to pass ULFS. A treatment causing such a relative decrease by which signs and symptoms become residual and the patients satisfied (‘much improved’ or better), could then be considered as being successful. Reversely, ULFS can be defined and subsequently a constant amount of relative decrease in score level which is required to pass ULFS, regardless of the patient’s baseline. Attaining functional status by this particular relative decrease will then yield a criterion for ending a patient’s treatment by the clinician as being potentially successful. This ending will then likely be related to the patient’s perception of, in this example, ‘much improved’ or better.

An index of relative change, ‘Treatment Duration Control’ (TDC) has been developed as a tool for clinicians to end or to continue a patient’s treatment in a randomized controlled trial in which treatment duration can vary. Like with a traditional ROM, the TDC-procedure yields data on treatment duration and number of visits needed. Furthermore, TDC, based on findings of a blinded assessor, yields data on success rate and therapy efficacy. The aims of the present paper are: (1) presenting the background of TDC, (2) showing its application to control treatment duration in patients with myogenous Temporomandibular Disorders in a way that concurs with clinical care, and (3) its validation. The present study involves TMD patients, but has potential for other chronic pain patients and even for other categories of patients for which perception of the degree of treatment effect is related to relative change in signs and symptoms. The TDC-criterion for a successful treatment will be validated by examining distributions of: (i) TDC-values, (ii) scores of intensity of the predominant pain in the oral system and (iii) utility values of Health-Related Quality of Life (HRQoL) being a variable that is entirely independent from TDC. It will be shown that: (i) sub-samples of patients in a bimodal distribution of TDC-values that occurred in the long-term, correspond largely with the patient groups having a successful and an unsuccessful treatment according to TDC; (ii) the group of patients with a successful treatment is associated with a distribution of scores of pain intensity that has become narrow following treatment and follow-up and consists of residual small values, while the distribution remains similarly broad in the group of patients with an unsuccessful treatment, (iii) the group of patients with a successful treatment is associated with scores of HRQoL that have much improved while the scores from patients with an unsuccessful treatment did not improve. The TDC-criterion for a successful treatment was further validated by data from the literature. First, the amount of relative decrease in the scores of pain intensity in TMD patients with a successful treatment was linked with an estimate of the patient’s assessment of the degree of improvement. This improvement was derived from the invariant association between relative decrease in pain intensity and the assessment of improvement for various types of chronic pain patients [15]. Second, the success rate of treatment according to the TDC-criterion was compared to success rates for myogenous TMD from the literature. A preliminary report on outcomes of therapies with variable duration for myogenous TMD, has been published previously [16].

## Methods

### Patients and general procedure

The study was carried out in compliance with the Helsinki Declaration, and approved by the University Ethics Committee (‘commissie Wetenschappelijk Onderzoek bij Mensen’, WOM, [committee for Scientific Research on Human subjects]) and the Board of Developmental Medicine (‘Ontwikkelingsgeneeskunde’, OWG); reference: OG/93/002. One hundred and eighteen patients with myogenous Temporomandibular Disorders, a chronic pain disorder, participated after providing informed consent. Appendix, section ‘Inclusion and exclusion criteria of the patients’ outlines the inclusion and exclusion criteria (for details, see also ref [17]).

Evaluation of a patient’s status was carried out not only by the person who carried out treatment (the ‘clinician’, a dentist for dental therapies and a physiotherapist for physiotherapy), but also by an assessor (another dentist) who was blinded to the type of treatment and the patient’s medical history. Using data from the assessor, a third dentist, the investigator (co-author RG), determined the outcome TDC-values for the randomized controlled trial, to keep the assessor blinded. All abovementioned persons were specialists in orofacial pain and Temporomandibular Disorders (TMD). When a physiotherapist carried out treatment, a dentist who was responsible for the patient, carried out a final evaluation as ‘clinician’.

The main characteristics of the procedure using relative change for a Randomized Controlled Trial with myogenous TMD patients, were:

1. Baseline scores from anamnestic and clinical items were obtained by a blinded assessor, just before treatment and transferred by the investigator to keep the assessor blinded;

2. Items with sufficiently large score values at baseline (i.e. score value of at least the smallest detectable difference, SDD. in the short term) were selected as basic reference items for monitoring relative change using the index TDC during treatment (by the clinician) and during follow-up (by the investigator, based on data from the blinded assessor). Thus relative change was tested adaptively only for those signs and symptoms which were pronounced.

3. Reference items of which relative change was monitored, could be added during treatment (based on data from the clinician) or following treatment (by the investigator, based on data from the blinded assessor) if their scores increased from a low level to a high level (from below SDD in the short-term to above SDD in the long-term). Possibly added reference items from the clinician contributed together with the basic reference items to the TDC-value on which the clinician’s decision was based when to end treatment. However, possibly added reference items from the clinician were ignored for determining post-treatment TDC-values so that they were solely based on data from the blinded assessor. The procedure of separately added reference items allowed, like in clinical care, monitoring of late pronounced signs and symptoms and provided data on success rate and efficacy of treatment which were not biased by the clinician or by inter-patient differences in treatment duration or number of visits.

4. The following option has been added to comply with usual clinical care and for ethical reasons: The patient’s opinion as reflected in anamnestic items on daily functioning of the oral system was given priority in the treatment outcome if the index of overall relative change (including changes related to items from clinical tests) indicated a ‘successful’ treatment while the anamnestic items alone indicated an ‘unsuccessful’ treatment.

### Background of TDC

**Figure 1:**
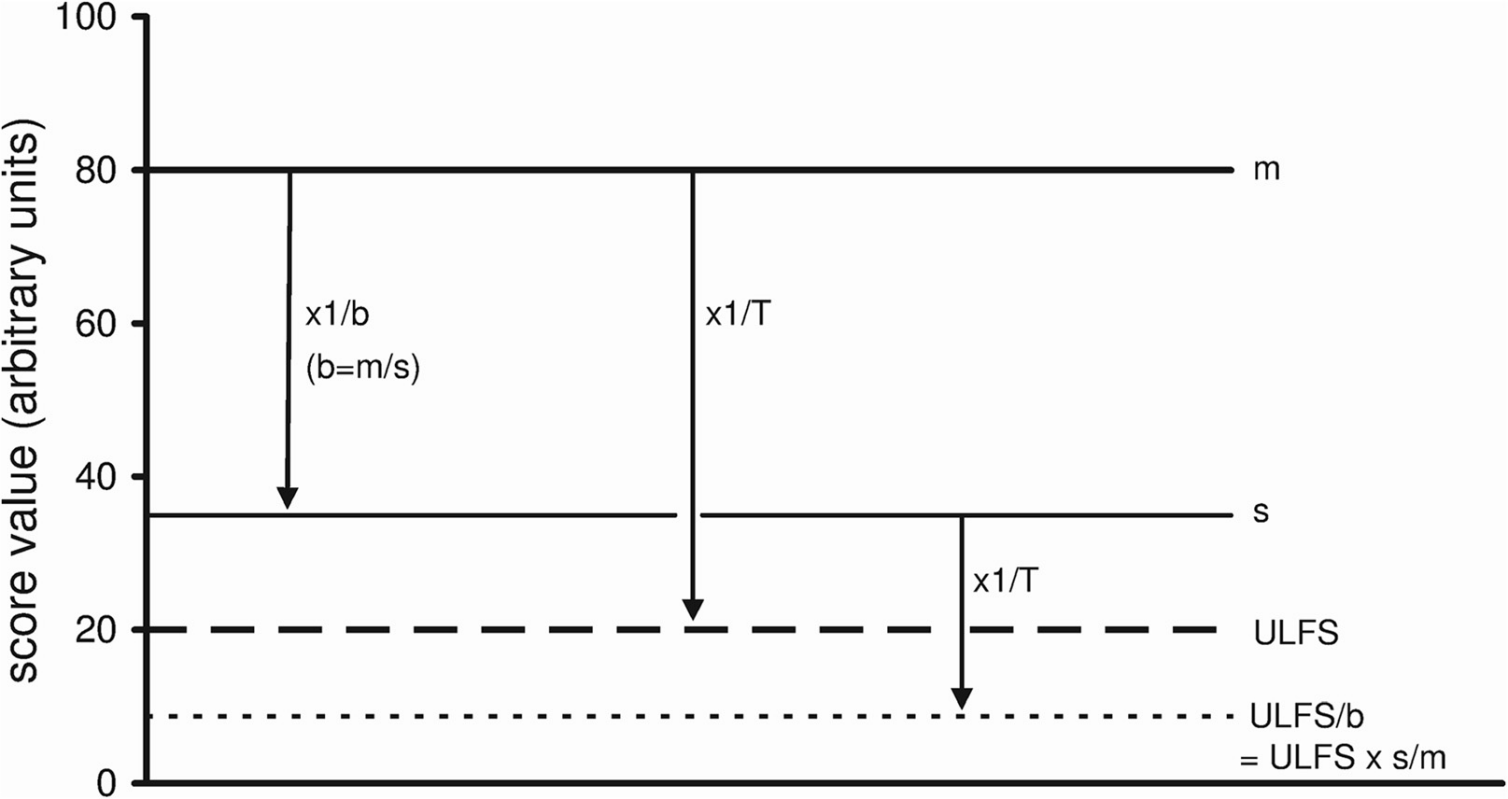
**Score levels of two patients, with a maximal baseline ‘*m*’ and a smaller baseline ‘*s*’ respectively.** ’*ULFS*’, upper limit of functional status. The zone of functional status with residual score levels is located between zero and *ULFS*. *T*, treatment factor by which the maximal baseline ‘*m*’ is just decreased to level *ULFS* (by a factor 4 in this example; *m* decreases from 80 to 20 units). When the same factor *T* is applied to the smaller baseline ‘*s*’of 35 units, this baseline is decreased below *ULFS* to *ULFS/b*, from 35 to 8.75 units. If factor *T* is tuned to the patient with baseline ‘*m*’, for reaching *ULFS*, the end level of any smaller baseline will enter the zone of functional status when the same factor *T* is applied to this smaller baseline. Mathematical proof: For the patient with level *m*, treatment must be so effective that *m* decreases at least by a treatment factor *T* to reach *ULFS*, thus: *m/T = ULFS* (*T > 1*) [equation (1)]. The ratio between the patients’ baselines equals *m/s = b* (*b*, baseline factor, *b > 1* ). Thus *m = s.b* and substituting *s.b* for *m* in equation (1) yields: *(s.b)/T = ULFS*, thus the score level reached by factor *T* for the patient with baseline *s* is given by: *s/T = ULFS/b*. Level *ULFS/b* (*b* > 1) is lower than level *ULFS*. Therefore, the value of factor *T* derived from a patient with the highest score level is applicable to any patient with a lower level for attaining a final level that falls within the zone of functional status. The end level (*ULFS/b*) for the patient with initially level ‘*s*’ equals (*ULFS.s/m*). Thus an end score will be located between zero and *ULFS*, proportionally with the baseline level ‘s’.

The use of relative change in score levels enables defining a constant factor for attaining functional status. Figure 1 depicts score levels of two patients, one with a maximally large baseline level ‘*m’* (for example of pain intensity), and another patient with a smaller baseline ‘*s’*. Functional status is related to a zone with low score levels between 0 and an Upper Limit of Functional Status (*ULFS*). Functional status in myogenous Temporomandibular Disorders is characterized by a low level of signs and symptoms of pain and impairment of the oral system that might occasionally occur in healthy subjects [17]. It is likely (see Background, Discussion) that attaining such a condition following treatment will be concomitant with a patient’s assessment of ‘much improved’ or better. In order to attain functional status for the patient with level *m*, this level should decrease to at least *ULFS*. Such a decrease will occur in a relative sense if treatment is so effective that level *m* is decreased by the ratio between *m* and *ULFS*, further denoted as the treatment factor ‘*T’* (thus *T* = *m*/*ULFS* and *m* decreases to *ULFS* by multiplying *m* with *1/T*). Figure 1 shows graphically that when a smaller baseline level ‘s’ of another patient is decreased by the same factor *T*, the zone of functional status is also attained for that patient, i.e. its post-treatment level drops below *ULFS*. Mathematically it follows that factor *T* derived from a patient with the largest score level is applicable to any patient with a smaller level (see legend of Figure 1). Furthermore, the end level is proportional to the baseline.

So far, factor *T* applies to a single score with levels ‘*m*’ and ‘*s*’. However, a disease or disorder includes a variety of signs and symptoms. On a particular type of scale, the scores related to various signs and symptoms have to decrease to a similar low score value before a treatment can be considered as being successful. Because in chronic pain patients, the assessment of degree of improvement by treatment is related to relative change in pain intensity, relative change will be relevant for any sign and symptom that is associated with pain. Myogenous TMD patients are suffering from chronic pain, mainly in facial areas, which is not caused by somatic disease [17]. All items from the anamnestic and clinical examination in the present study were related to intensity or frequency of pain from the masticatory system, and to functioning of the oral system in daily use and in clinical tests, which was impaired by the presence of pain. Because of this general association with pain, relative change from different items was equally weighted for deriving a measure of global relative change. Such a weighting is further supported by the finding that the expectation of patients with facial pain or fibromyalgia regarding treatment of their symptoms is constant in a relative sense [14]. This expectation of relative reduction of signs varied within a small range from 56% to 63%, regardless of the domain of scoring (pain, fatigue, distress or interference with daily activities) or the patients’ background (type of chronic pain, baseline level).

In order to consider all relative changes in the recovery of individual patients, an overall factor of change has been derived from all score changes within patients. Since ratio values between two successive measurements lack an appropriate zero point to attain a meaningful arithmetic mean (the ‘usual’ mean), such values were transformed as Contrast-values. Contrast-values have a zero point to which values of an equivalent relative increase and decrease have the same distance (Appendix, section ‘Averaging of ratio values between scores from two times of measurement’). The Contrast, *C*_*i*_ between two measurements of item *i* is given by:

$$C_{i}=\left(S_{2,i}-S_{1,i}\right)/\left(S_{2,i}+S_{1,i}\right),$$

in which *S*_*1,i*_ is the score of the *i*-th item at a first visit (the ‘reference’ visit), and *S*_*2,i*_ the score at a later visit.

Thus Contrast, being the ratio between difference and sum, is a normalized difference between two measurements. When there is no change (S_2,*i*_ = S_1,*i*_), C_*i*_ is zero. When signs or symptoms related to item *i* disappear (S_2,*i*_ = 0), C_*i*_ has the value of −1 [= (0 - *S*_*1,i*_)/(0 + *S*_*1,i*_)]. If signs or symptoms worsen, C_*i*_ has a positive value (maximum: +1). Thus the possible Contrast-values vary within a range from −1 to +1.

All patient’s Contrast (C_*i*_) values were averaged for each visit during treatment or follow-up, yielding a single index, ‘Treatment Duration Control’ (TDC), related to global relative change, thus:

$$\mathrm{TDC}=\left(\sum_{i=1}^{n}C_{i}\right)/n,$$

in which *n* is the number of items.

A cut-off point of TDC is related to an overall value of the treatment factor *T* required to attain functional status across several items. As for factor *T* of a single score, data from a patient with overall maximal signs and symptoms (from pilot data, see below) have been used to derive the overall factor *T* which is related to all scores changes within that patient, and which is required to attain the upper limit of functional status. An overall change by *T* yields then a criterion for ending treatment in any patient in the usual way of clinical care, i.e. by attaining functional status across several signs and symptoms (Figure 1).

For two reasons, the use of a patient with maximal signs and symptoms is appropriate to derive an overall value of *T* for all patients. First, an accurate assessment of an overall factor *T* within a patient is only possible when a sufficient number of scores is available with large values so that 0–4 point scales (used in the present study, see below) are nearly entirely used. A patient with overall maximal signs and symptoms had many scores (n = 32 for the patient from the pilot data) with, in general, large values. Second, following a change by an overall factor *T* derived from the patient with maximal signs and symptoms, the end levels of any patient will, on average, be proportional to the base line (Figure 1). Such end levels concur with the empiric relationship between relative decrease of pain intensity and assessment of treatment effect that is independent from pain intensity at baseline ([15], *cf.*Discussion).

In order to control the duration of treatment, two cut-off values of the index TDC are necessary to comply with clinical care. A first cut-off point (related to a smaller factor than the overall factor *T*) serves to decide whether a patient has responded sufficiently following a treatment-specific time interval. If not, the clinician can stop this treatment. Second, a cut-off point related to factor *T*, serves to decide whether the upper limit of functional status has passed and the treatment has become potentially ‘successful’. Treatment can then be ended before a preset upper limit of treatment duration is exceeded.

The cut-off points of TDC in the present study were based on scores on extent and/or frequency, using adjectival 0–4 point scales (Table 1) for items which were related to pain or impairment of oral functioning. These scores were obtained during the anamnestic and clinical examination of the TMD patients. An anamnestic questionnaire included 5 scores of items related to daily oral functioning, and the clinical examination included 42 scores of pain intensity during movement and clenching tests and muscle palpation (Appendix, section ‘Scores from anamnesis and clinical examination’). The cut-off points of TDC derived from scores from the 0–4 points scales were also valid for ratios in subsequent scores of the intensity of the predominant pain in the masticatory system from a 100 mm Visual Analogue Scale (VAS; part of anamnesis, see Appendix). A generalized use of cut-off points of TDC is supported by the finding that in normalized form, clinically relevant changes in scores of different items are similar for myogenous TMD, regardless of the type of scale used [18].

**Table 1:** Adjectival 0–4 point scales for pain intensity, frequency of pain and frequency of impaired function

**Score value**	**Intensity of pain**	**Frequency of pain**	**Frequency of impairment**
0	no pain	never painful	never impairment
1	slight pain	sometimes painful	sometimes impairment
2	moderate pain	regularly painful	regularly impairment
3	severe pain	often painful	often impairment
4	extreme pain	permanently painful	permanently impairment

Appendix, section ‘Choosing two cut-off points of TDC’, explains how the two cut-off points were chosen for TDC. The first cut-off point was TDC = −0.212, which corresponds to a decrease of 35% in a single score of pain intensity at a 100 mm VAS [−0.212 = (65 – 100)/(65 + 100)]. Three decimals are used to have negligible rounding off errors when *C* or TDC-values are transformed back. If a patient’s TDC was larger than −0.212 (TDC > −0.212) at a critical stage of treatment, the patient was insufficiently responsive to treatment. A less negative value than −0.212 means less change towards recovery (note that TDC = −1 with zero signs or symptoms left). The second cut-off point, TDC = −0.379, was related to attaining functional status (‘successful’ treatment), and corresponds to 55% decrease of a single score of pain intensity (−0.379 = (45 – 100)/(45 + 100)). As outlined in Appendix, this second cut-off point was based on baseline scores from a patient with overall maximal signs and symptoms in a pilot sample of 20 patients, and on a panel opinion regarding the Upper Limit of Functional Status, *ULFS*, across various items. When TDC was ≤ −0.379, treatment became potentially successful.

Before treatment is started, the score values of the various items might vary between low and large values. In traditional Routine Outcome Monitoring, all scores and their changes during treatment are included in the multi-dimensional questionnaire used. However, for the TDC-procedure, it is important to select basic ‘reference’ items that contribute substantially to Contrast-values and TDC. Score values have a limited accuracy which is reflected in the statistical value of the Smallest Detectable Difference (SDD). Some changes might therefore be based on chance fluctuations. Although the raw change is small between successive scores which are both small, the relative change between such scores might be even larger than the relative change between two score values of which one is large. As a numerical example with scores from a 0–4 point scale: the relative change between the starting and subsequent score values ‘1’ and ‘0’ yields an extreme Contrast-value of −1 (= (0 – 1)/(0 + 1)) while a Contrast-value of −0.500 occurs when a score of ‘3’ decreases to ‘1’ (−0.500 = (1 – 3)/(1 + 3)). However, even the largest possible raw decrease towards zero of the score value ‘1’ from the pair ‘1’ and ‘0’ (a maximal decrease of 1 unit), might be solely due to chance fluctuations because a decrease of 1 unit is smaller than an SDD value of, for example, 2 units. Including such insignificant changes as Contrast-values in TDC would create noise components that would mask the effect of relative decreases in pronounced signs and symptoms that reflect improvement due to treatment. The value of SDD can be used as a threshold for selecting reference items with a sufficient large starting value, i.e. their maximally possible decrease towards zero should exceed SDD [18].

Items scored on 0–4 point scales were selected before treatment if their baseline exceeded the SDD of a single score for a test-retest interval of one week. This SDD is 1.9 units (46.8% of the scale range [18]). Thus ‘basic reference items’ had a baseline of at least 2 units (corresponding to at least ‘moderate pain’, ‘regularly painful’, or ‘regularly impaired function’; Table 1), and were related to a patient’s pronounced signs and symptoms. The intensity of the predominant pain in the masticatory system, scored on a 100 mm Visual Analogue Scale, was also a basic reference item.

In common clinical care it is usual that a clinician follows all pronounced signs or symptoms, including ones that might be insignificant at baseline but become pronounced during treatment. In traditional Routine Outcome Monitoring or a traditional Randomized Controlled Trial, the increased scores of such late pronounced signs or symptoms are automatically included in the multi-dimensional questionnaire used. If such scores remain large in ROM they might ultimately contribute to an increased overall outcome score and hence to a decreased success rate and efficacy of therapy. In order to allow monitoring such late pronounced signs or symptoms in the TDC-procedure, reference items could be added during a visit following the baseline measurements. It is then of interest (cf. Discussion) to avoid possible bias in the TDC-related outcome variables success rate and treatment efficacy, which might be clinician-bound or might be due to inter-patient differences in the number of visits or in duration of treatment. To that end, reference items that were added during treatment by the clinician were separately considered from those added following treatment on the basis of data from the blinded assessor (details, see below). As an example of addition: suppose that an item has a score value of ‘1’ before treatment and that this score increases to ‘3’ during treatment. The increase to score level ‘3’ is relevant because a potential decrease of 3 to 0 (3 units) during subsequent treatment is larger than SDD.

Based on scores of the clinician, reference items were added to control treatment duration appropriately, if the patient’s scores increased during treatment from a pre-treatment level of ‘0’ or ‘1’ (a low severity level, i.e. at most ‘slight’ pain, ‘sometimes’ painful, or ‘sometimes’ impairment; Table 1) to ‘3’ or ‘4’ (a high severity level, i.e. at least ‘severe’ pain, ‘often’ painful or ‘often’ impairment). A threshold of 3 units for the maximally possible decrease from a score ‘3’ towards zero, exceeds the long-term SDD of a single score being 2.2 units (54.5% of the scale range [18]). In order to minimize the influence of chance fluctuations in the addition procedure, the long-term SDD value was chosen as a slightly more conservative criterion than the short-term SDD of 1.9 units used for selecting basic reference items. The first time an item *i* was added as a reference, its Contrast-value (C_*i*_) was calculated using the low pre-treatment score value as a base-line (S_1,*i*_ in equation (1)) on this occasion. For example, a pre-treatment score was ‘1’, while a score of ‘3’ was observed for the first time during a later visit. The C_*i*_ value was then +0.500 [= (3 – 1)/(3 + 1)], in which the positive sign reflects an increased severity of the added item for this particular visit. The increased score value (‘3’ in this example) was used as the reference level (S_1,*i*_ in equation (1)) for subsequent visits to describe any relative change of severity (decrease or increase) with respect to the visit of addition (the ‘reference visit’).

TDC is primarily used as a control variable that signals to the clinician that a patient has entered the zone of functional status. The amount of relative decrease required to pass the upper limit of this zone, has been defined *a priori*, and is thus constant. The precise value of TDC at the end of treatment is not of interest for a clinician’s decision of a potentially successful treatment but meeting the criterion TDC ≤ −0.379, for sufficient relative improvement which applies to any patient, is. Worsening signs and symptoms related to the addition of reference items means that the general level of a patient’s reference scores will increase somewhat. An increased score level of basic reference items might also be involved in this general increase at the stage of addition, yielding an increase of the general severity level of TMD. If the possible treatment duration has not expired and the patient is further responsive, such a patient will still be able to attain and pass the upper limit of the zone of functional status as long as the general score level will remain below that of the patient with maximal baseline values to which the cut-off point TDC = −0.379 has been tuned *a priori*. In accordance with clinical care, the increase in severity level of myogenous TMD, to which the addition of reference items is related, may extent the duration of treatment, even when this increase is temporarily. More visits are then required before a clinician can decide, using the TDC-criterion (TDC ≤ −0.379), that a treatment has become potentially successful. When an increase in score value is sustained and a basic reference item is not involved, the item with the sustained larger score during treatment will likely also have a large score value in the data from the assessor following treatment. This large score will then be detected as a post-treatment added reference item. Apart to contributing to a possible decrease in success rate, a sustained increased score will then yield an increase in the post-treatment TDC-value and thus tend to decrease the efficacy of the therapy at a group level. The effect of addition of reference items on success rate and therapy efficacy will be shown be comparing in retrospect these parameters between different modes of addition including the mode without addition.

One might argue that selecting basic reference items and added reference ones by using a threshold of score values might introduce a bias in the treatment outcome which is due to regression to the mean. Large score values will tend to decrease rather than to increase by chance alone. If treatment success and efficacy were solely based on raw score values with a selection threshold, these parameters of treatment outcome would be overestimated, particularly in patients with large baseline values. However, chance effects are neutralized when the criterion for a successful treatment is based on a constant amount of relative change rather than on criteria which are related to raw change. In the TDC-procedure, patients should have proportionally more raw decrease in the score values of their selected reference items for attaining functional status, the larger their baseline values are. Mathematically it follows that bias by regression to the mean is lacking in relative decrease of any item that contributes to TDC, in particular when Contrast-values are used (Appendix, section ‘Lack of bias by regression to the mean in Contrast and TDC-values’). A lack of regression to the mean for relative change was further demonstrated using data from the present study. The relationship between raw difference in post-treatment and baseline scores of pain intensity, and baseline scores of pain intensity was examined as an example in which regression to the mean is involved. The relationship between the Contrast of pain intensity and baseline pain intensity was examined to show that the use of Contrast-values of pain intensity eliminated any regression. The absence of regression was further verified by examining the relationship between post-treatment TDC-values and the baseline of two variables that were related to the severity of myogenous TMD: (i) the intensity of the predominant pain in the oral system and (ii) utility values of Health-Related Quality of Life.

Following the introduction of all score values in a custom-made spreadsheet ((Microsoft Excel®; available on request) the reference items (including added ones) were automatically detected and Contrast and TDC-values were automatically determined for each patient and the various visits. Table 2 shows a patient example of Contrast and TDC-values.

**Table 2:** Patient example of contrast-values and the index ‘Treatment Duration Control’ (TDC)

**Reference item (*i *)**	**Reference score (S**_**1,** ***i***_**)**	**Later score (S**_**2,** ***i***_**)**	**Contrast,** ***C***_***i***_ ***=*** **(S**_**2,** ***i***_**- S**_**1,** ***i***_**)/(S**_**2,** ***i***_**+ S**_**1,** ***i***_**)**
*anamnesis:*			
(1) VAS-score of intensity of predominant pain (mm)	20	3	−0.739
(2) pain of the jaws (frequency)	3	1	−0.500
(3) stiffness and/or fatigue of the jaw muscles (frequency)	3	1	−0.500
(4) impaired movement of the jaw (frequency)	3	0	−1.000
			
*clinical examination:*			
(5) pain intensity on the right side during passive jaw opening	2	0	−1.000
(6) pain intensity on the left side during passive jaw opening	2	0	−1.000
(7) pain intensity during palpation of the right deep masseter muscle	2	1	−0.333
(8) pain intensity during palpation of the left deep masseter muscle	2	1	−0.333
(9) pain intensity during palpation of the insertion of the right occipital muscle	2	0	−1.000
(10) pain intensity during palpation of the insertion of the left occipital muscle	2	1	−0.333
			
TDC_anamnestic-items_ = (∑ C*i*)/4 = [−0.739-0.500-0.500-1.000]/4) =			−0.685
TDC_clinical-items_ = (∑ C*i*)/6 = 1.000-1.000-0.333-0.333-1.000-0.333]/6) =			−0.666
TDC = (∑ C*i*)/10 = [−0.739-(2 × 0.500)-(4 × 1.000)-(3 × 0.333)]/10) =			−0.674

Reference item (*i*), item with a sufficiently large score-value (see text) of which changes are followed. In this example of a patient, there are 4 reference items related to anamnestic questions and 6 items related to the clinical examination, thus 10 reference items in total (*i = 1..10*). *S*_*2,i*_, score value of item *i* at a later visit (‘visit 2’) than *S*_*1,i*_, the reference value item *i* that was observed for the first time at an earlier visit (‘visit 1’). Except the VAS-scores, all other scores originate from adjectival 0–4 point scales (Table 1). *C*_*i*_, Contrast-value being the ratio of the difference and sum between the second and the first score values of item *i*.. TDC, the index Treatment Duration Control, being the mean Contrast-value averaged across all items. TDC_anamnestic-items_ and TDC_clinical-items_, mean TDC averaged across the anamnestic items and the clinical items respectively.

### Pre-treatment procedure

After diagnosis, the patients were randomly allocated within two pairs of therapies, i.e. (1) occlusal splint (n = 35) versus physiotherapy of the masticatory system (n = 37), and (2) occlusal adjustment (OA; n = 23) versus a combination of occlusal splint and OA (n = 23). Conventional dental therapies include splint and/or OA.

The preset lower and upper limits for the number of visits and the treatment duration varied between the various types of therapy, with a total range of 3–15 (visits) and 6–30 weeks (duration).

The blinded assessor carried out an anamnestic and a clinical examination just before the start of a patient’s treatment to obtain baseline scores of TMD signs and symptoms (Table 3, stage 2). Using these data, a list of basic reference items was prepared by the investigator before treatment was started.

**Table 3:** Procedure of a randomized controlled trial with variable treatment duration, for myogenous TMD

**Stage 1**	**Stage 2**	**Stage 3**	**Stage 4**	**Stage 5**
intake randomization	pre-Tx anamnestic and clinical data from blinded assessor	anamnestic and clinical data from clinician	post-Tx anamnestic and clinical data from blinded assessor	follow-up: anamnestic and clinical data from blinded assessor
	list of baseline reference items from investigator (based on data from assessor)	TDC from clinician, using list of investigator (data from assessor), and possibly based on added reference items from clinician	TDC from investigator based on assessor’s data; short-term outcome of RCT: if TDC ≤ -0.379 and no *application of discrepancy rule from anamnesis: Tx successful for short-term RCT-outcome, and patient continues to follow-up (stage 5); otherwise, Tx unsuccessful	TDC from investigator, based on assessor’s data; long-term outcome of RCT: if TDC ≤ -0.379 and no *application of discrepancy rule from anamnesis: Tx successful; otherwise, Tx unsuccessful
	start of Tx by clinician	if TDC > -0.212 (after Tx-specific minimal Tx-duration), Tx ended unsuccessfully (insufficiently responsive patient), patient referred to blinded assessor (stage 4)	**if TDC from clinician > -0.379, while TDC from investigator ≤ -0.379 and no *application of discrepancy rule from anamnesis: Tx successful for short-term RCT-outcome, and patient continues on observation for follow-up (stage 5; TMD, not life-threatening)	
		if TDC ≤ -0.379, at two successive visits (interval of 3-6 weeks) and no *application of discrepancy rule from anamnesis: Tx successful according to clinician, patient referred to blinded assessor (stage 4)		
		otherwise, Tx continued if the pre-set maximal treatment duration is not exceeded; then patient referred to blinded assessor (stage 4)		

Stage 3, period of treatment (Tx) with a Tx-specific range of possible Tx-duration. Stage 5, follow-up, half a year and a year respectively following the end of a treatment that is successful in the short-term (stage 4). TDC, the index ‘Treatment Duration Control’. TMD, Temperomandibular Disorders. *application of discrepancy rule from anamnesis: if overall TDC ≤ -0.379 (successful Tx) while TDC solely based on anamnestic items >-0.212 (Tx with insufficient effect according to the patient), Tx was considered as being unsucessful. **this rule needed not to be applied in the present study as a discrepancy did not occur between a TDC-based outcome on treatment success from the clinician and the investigator (based on the assessor’s data). For further explanation, see text.

The anamnestic questionnaire included, apart from a VAS-score of the intensity of the predominant pain from the masticatory system, scores on 0–4 point scales of other items related to daily oral functioning (Table 1; 6 items in total; Appendix, section ‘Scores from anamnesis and clinical examination’). The clinical examination included scoring of pain intensity during movement and clenching tests and muscle palpation (42 items). By placing Table 1 in his or her sight, the patient could tell the score number or indicate it by finger signaling, limiting time load by the clinical examination to 15–20 minutes.

### Treatment procedure

The clinician carried out the same anamnestic and clinical examination as the assessor at various visits (Table 3, stage 3). For determining TDC, the clinician not only considered the basic reference items but actually increased score values could also yield added reference items (see above, section ‘Background of TDC’).

Patients expressed the daily functioning of the oral system by means of anamnestic reference items whereas clinicians expressed the functioning of the oral system in clinical tests by clinical reference items. Patients assessed a smaller degree of improvement at the end of treatment than clinicians (cf. Results). The patient’s opinion was therefore given more weight if the outcome from the anamnestic items indicated a demand for further treatment, by application of the following ‘discrepancy rule’. If the overall TDC was  ≤ −0.379 (successful treatment), but TDC-anamnestic-items was > −0.212 (treatment with insufficient effect according to the patient), the treatment was considered as unfinished or as being unsuccessful if the maximal therapy duration was exceeded.

Depending on the TDC-outcome, the clinician continued or finished treatment within preset limits of possible therapy duration. If TDC was > −0.212 after a treatment-specific minimum duration of treatment, the treatment was ended because the patient was not sufficiently responsive. If −0.379 < TDC ≤ −0.212, a patient was sufficiently responsive but the treatment was continued. If TDC was ≤ −0.379 at two successive visits with a therapy-specific interval of 3–6 weeks, while the discrepancy rule was not applied, treatment was ended as being potentially successful.

### Outcome procedure

The assessor recorded the scores, on average 4.8 weeks (SD 4.7) after the end of treatment for all patients, and after 6 and 12 months of follow-up for those patients whose treatments were successful in the short-term (Table 3, stage 4 and 5). Patients with an unsuccessful treatment in the short-term had no follow-up, because their initial treatment had to be stepped up or changed for ethical reasons and in accordance with clinical care.

The investigator determined the TDC-value for each patient using the patient’s basic reference items. Furthermore, those items were added as a reference of which the assessor’s score had increased from a level of ‘0’ or ‘1’ at baseline to a level of ‘3’ or ‘4’ at a post-treatment visit. Possibly added reference items from the clinician were ignored to obtain success rates of treatments and post-treatment values of TDC related to therapy efficacy that were solely based on data from the blinded assessor. Furthermore, by considering the treatment period as a black box, any bias is avoided in the post-treatment TDC-values which might be due to inter-patient differences in the number of visits during treatment or in the duration of treatment (cf. Discussion).

Success rate of myogenous TMD (occasionally corrected by the aforementioned discrepancy rule) was determined in the entire patient group, as no significant differences occurred between therapy types.

### Validation of cut-off points of TDC

The cut-off TDC = −0.379 was validated by considering distributions of TDC-values, VAS-scores of pain intensity, and utility values of EQ-5D [19] related to Health-related Quality of Life (HRQoL). The cut-off points TDC = −0.202 and TDC = −0.379 were validated by data from the literature.

### Statistical analysis

Statistical analyses were performed using Graphpad software (Graphpad Prism 6.01, Graphpad Software Inc, San Diego, CA). For each therapy, TDC based on anamnestic items was compared with TDC from clinical items, in two-way ANOVAs for paired observations. These TDCs were compared at three occasions of treatment evaluation: (1) the last visit of treatment (‘pre-end-measurement’ PEM; clinician involved), (2) the visit to determine treatment outcome in the short-term (‘end-measurement’ , EM; assessor involved), and (3) the visit to determine the ultimate treatment outcome, finishing follow-up (‘last-measurement’ , LM; assessor). As EM was also LM for those patients whose treatment was unsuccessful at EM, 24.6% of the data was common between EM and LM. Two separate ANOVAs were therefore applied to compare TDC from PEM with that of EM and LM respectively. When ANOVA was significant at a level of 2.5% (Bonferroni correction of 5% for the twofold use of data), Bonferroni’s multiple comparison tests were used to determine significant differences between each pair of results.

A separate possible addition of reference items by the clinician during treatment and by the investigator (based on data from the assessor) during follow-up, and considering only the added items from the assessor, was the standard procedure for determining success rate and post-treatment TDC-values related to therapy efficacy. In order to assess the effect of addition, this mode was in retrospect compared with two other modes, i.e. (i) a mode of continual addition in which items are possibly added by the clinician and subsequently by the investigator (based on data from the assessor), are considered, and (ii) a mode without addition, in which only the basic reference items are considered which were obtained before treatment was started. Frequencies of patients including those related to success rate were compared between different conditions in a chi-square test. Two separate one-way ANOVAs for paired observations were applied to compare the TDC-values (pooled across therapies) between the three modes of addition at the two post-treatment occasions of treatment evaluation, EM and LM.

Regression analysis was applied to the relationship between TDC and baseline values of the intensity of the predominant pain and Health-related Quality of Life respectively to examine whether TDC depends on baseline values of variables that are related to the severity of myogenous Temporomandibular Disorders.

Wilcoxon’s test for paired observations was used to examine the significance of differences between pre- and post-treatment VAS-scores of pain intensity and utility values of EQ-5D.

## Results

### TDCs based on anamnestic and clinical items

**Figure 2:**
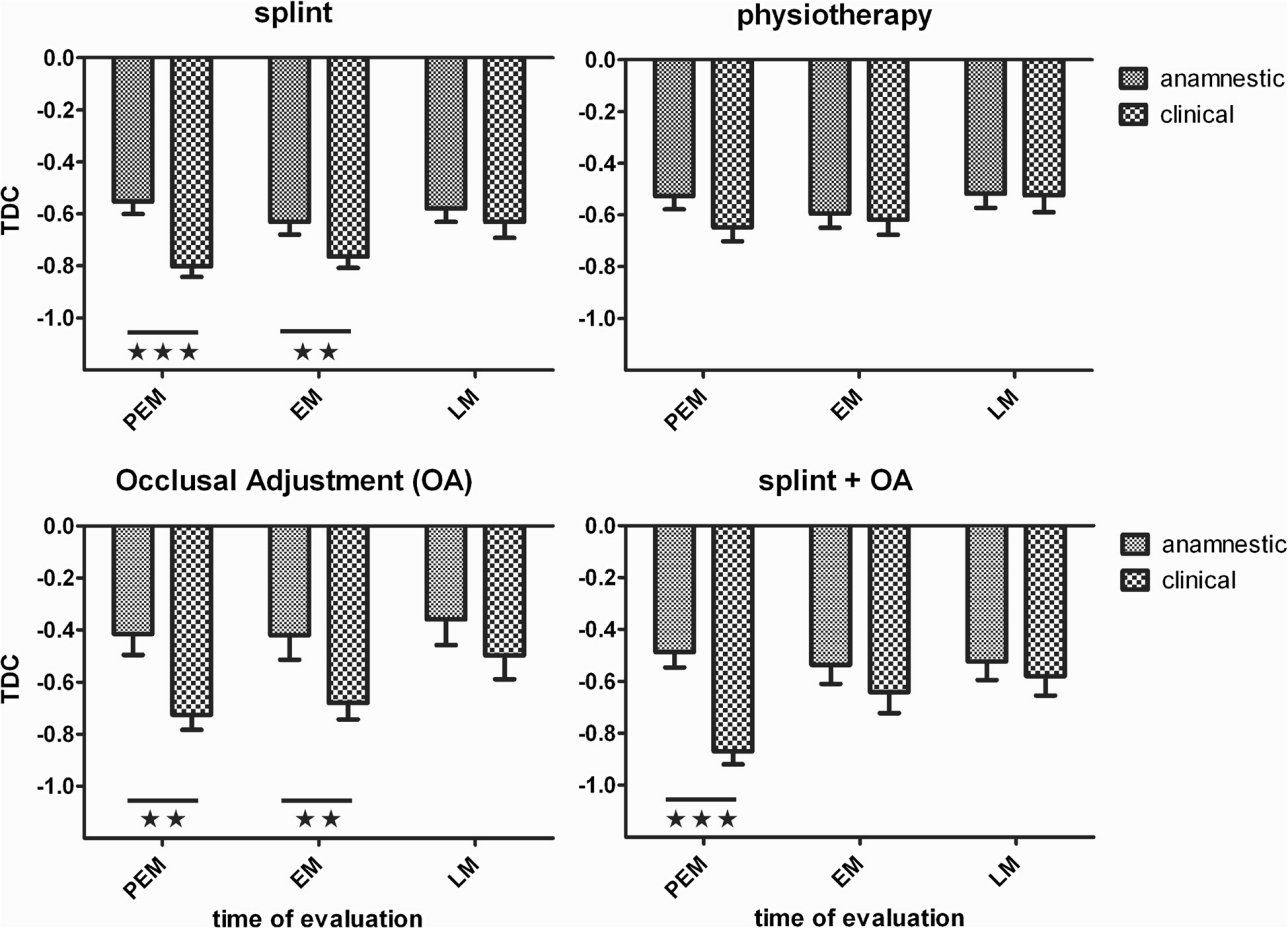
**TDC-values (mean and SEM) based on anamnestic and clinical reference items respectively (four therapies).** Three occasions of treatment (Tx) evaluation: (1) PEM, ‘pre-end-measurement’ of Tx-outcome, by the clinician at the last Tx-visit; (2) EM, ‘end-measurement’ of Tx-outcome in the short-term, by the assessor at the first post-Tx visit, and (3) LM, ‘last measurement’ with the ultimate Tx-outcome, by the assessor at the last post-Tx visit. TDC-values at PEM include the effect of possibly added reference items from the clinician and TDC-values at EM and LM from solely the assessor (cf. Table 4, mode s-A). Other modes of addition, including no-addition, yielded similar results (not shown here). The horizontal bars indicate cases of significant differences (Bonferroni’s multiple comparison tests) between TDC-anamnestic and TDC-clinical at various times of evaluation; **, p < 0.01; ***, p < 0.001. Significant differences between occasions of evaluation (not shown) occurred only for TDC-clinical, between PEM and LM for splint-Tx (p < 0.05) and for the combination of splint and occusal adjustment (splint + OA, p < 0.05).Note that the values of TDC-anamnestic were similar for the three occasions of evaluation. For the dental therapies, TDC-clinical increased (less negative TDC-values related to less improvement of the patients) at the post-Tx occasions so that TDC-clinical approached TDC-anamnestic at LM.

Figure 2 shows TDC-values related to anamnestic and clinical items respectively on three evaluation occasions (‘pre-end-measurement’ , PEM, at the last treatment visit; ‘end-measurement’, EM and ‘last-measurement’ , LM, both occasions following treatment). Two-way ANOVAs for repeated measures showed a significant (p < 0.001-0.01) effect of the type of TDC, for the three dental therapies. Bonferroni’s post tests showed that at PEM (involvement of clinician), TDC-anamnestic was consistently larger (p < 0.001-0.01; less negative values indicating less improvement) than TDC-clinical. Some significant differences occurred at EM and no significant differences at LM (involvement of assessor). The ANOVA was not significant for physiotherapy for which the evaluation was always carried out by another person than the physiotherapist, i.e. the responsible dentist at PEM and the assessor at EM and LM. However, TDC-anamnestic was significantly larger than TDC-clinical (p < 0.05; Student’s t-test for paired observations) at the visit before PEM, in which the physiotherapist was involved.

Differences between TDC-anamnestic and TDC-clinical did not depend on the level of TDC-values as regressions between the difference and the mean of paired values were non-significant.

### The influence of added reference items

The mean number of items that contributed to TDC was 14.2 at PEM (SD 8.0, range: 2–40, n = 118 patients), and 13.8 at LM (SD 7.5). Added reference items, based on data from either the clinician or the assessor were involved in 44.1% of the patients (n = 52). The clinician added reference items in 30.5% of the patients (mean 3.3 items, SD 3.7, range: 1–17, n = 36 patients) and the investigator (data from the assessor) in 27.1% of the patients (mean 2.5 items, SD 1.6, range: 1–7, n = 33 patients). A large majority of the reference items were basic reference items in the patients with added reference items. On average, 83.7% were basic reference items and 16.3% added reference items. Furthermore, addition of items occurred frequently in patients whose treatment was unsuccessful in the long-term (at LM), i.e. in 69.4% of the patients evaluated by the clinician at PEM and in 84.4% evaluated by the assessor at LM. The number of added reference items tended to be the largest for patients with a moderately large number of basic reference items (10–25 basic reference items). Addition needed never to be applied to patients with large numbers of basic reference items (clinician: >25; data from the assessor: >30). The summed score level, mean level, or the total number of scores from reference items of patients for which addition occurred, therefore never exceeded the values of the patient with maximal baseline values from a pilot sample, to which the cut-off point TDC = −0.379 has been tuned (see Appendix, section ‘choosing two cut-off points of TDC’). The current sample of 118 patients included 2 patients whose baseline values of summed score level, mean level and total number of reference items exceeded slightly those of the patient from the pilot sample (see also Appendix). Both patients had a successful treatment in the long-term.

**Figure 3:**
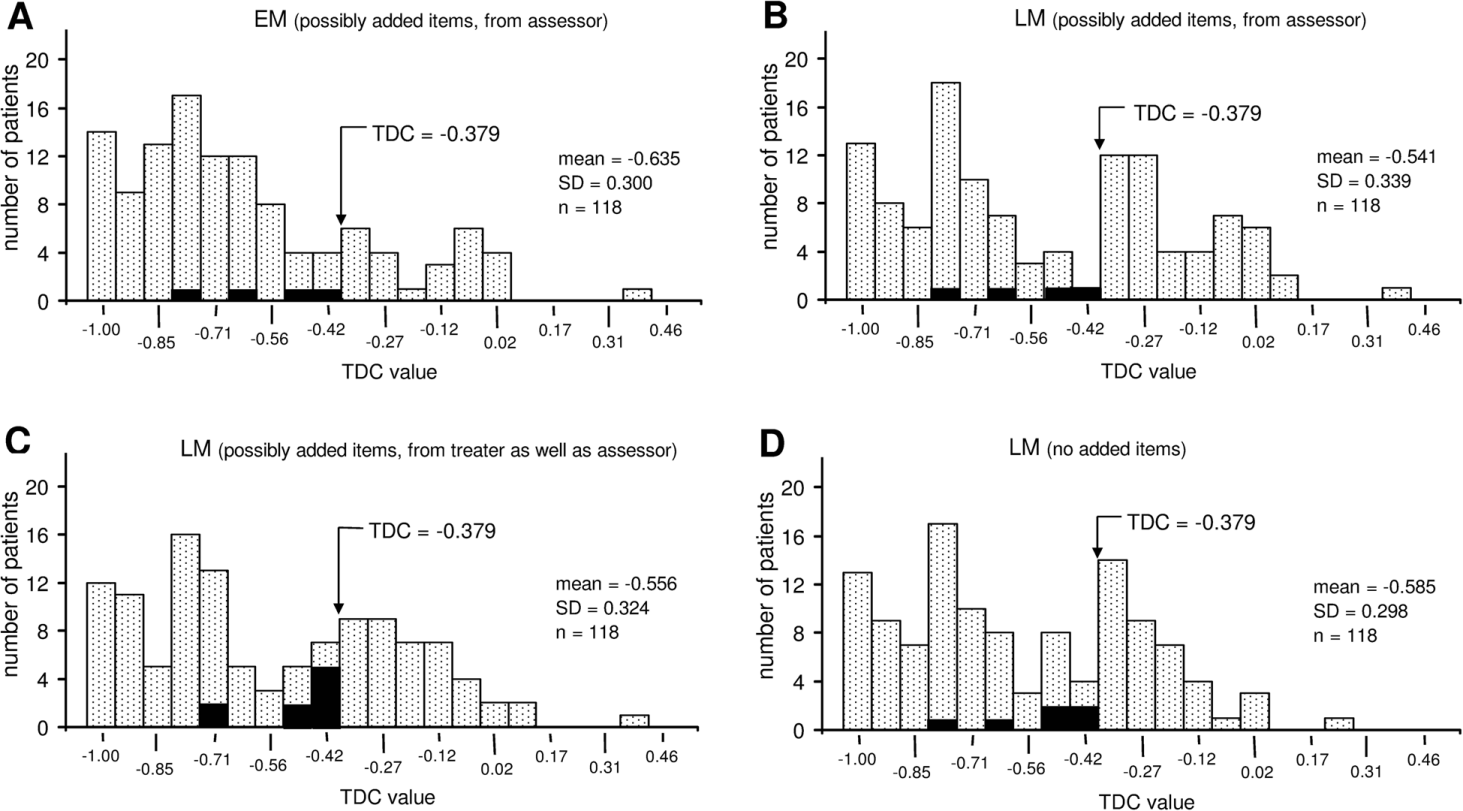
**Post-treatment distributions of TDC-values.** These distributions are depicted at two occasions of treatment (Tx) evaluation and with different modes of addition of reference items. Arrow, the cut-off point TDC = −0.379 for distinguishing between a successful Tx (more negative TDC-values to the left) and an unsuccessful Tx (less negative values to the right). Total number of patients: 118. **A-B**, the evolution of the TDC-distribution from the short-term to the long-term, post-Tx; possibly added reference items from solely the assessor were included in the TDC-values. **A**, TDC-distribution at EM (‘end measurement’, cf. Figure 2). **B**, ultimate TDC-distribution at LM (‘last measurement’). Note that the TDC-distribution became bimodal at LM. **C-D**, TDC-distributions at LM, with two other modes of addition of reference items: (1) items both from the clinician and subsequently the assessor **(C)**, and (2) no addition **(D)**. Note that, regardless of the way of addition, the three TDC-distributions at LM were bimodal **(B**, **C-D**) and that these distributions were similar. Black bars, patients (3.4-7.6%) whose treatments were successful according to the sole criterion of TDC ≤ −0.379, but unsuccessful according to the ‘discrepancy rule’ (see text, section ‘treatment procedure’). See Table 4 for the success rate at various times of treatment evaluation and various modes of addition of reference items, including the effect of application of the discrepancy rule. Occasions of evaluation and modes of addition in this figure **(A-D)** corresponds with EM, s-A / LM, s-A / LM, c-A and LM, NA respectively in Table 4.

Figures 3A-B, shows distributions of TDC-values in which only the added reference items from the assessor were considered at EM and LM. This distribution became bimodal at LM. Similar, also bimodal TDC-distributions occurred at LM when the added reference items from both the clinician and the assessor were considered, and when no items were added (Figures 3C-D).

Table 4 shows the effects of three modes of addition on success rate of treatment. Separately added items from clinician and assessor, and only considering those from the assessor, yielded, with the least frequent use of the discrepancy rule (3.4% of the patients), the most conservative success rate at LM. The differences between modes were, however, small and non-significant in chi-square-tests.

**Table 4:** Effect of added reference items and the discrepancy rule on the success rate of treatment

**Occasion of evaluation**	**Mode of adding reference items**	**n TDC ≤ −0.379**	**n TDC > −0.379**	**n discrepancy rule**	**n S-Tx**	**n U-Tx**	**Success rate (%)**
PEM	A	100	18	8	92	26	78.0
PEM	NA	100	18	8	92	26	78.0
							
EM	s-A	93	25	4	89	29	75.4
EM	c-A	96	22	8	88	30	74.6
EM	NA	98	20	6	92	26	78.0
							
LM	s-A	70	48	4	66	52	55.9
LM	c-A	78*	40*	9*	69*	49*	58.5*
LM	NA	79*	39*	6*	73*	45*	61.9*

Occasion of evaluation of treatment (Tx) success rate: PEM (clinician); EM, (assessor); LM, (assessor; cf legend of Figure 2). Mode of adding reference items: A, added (by clinician); s-A, separately added by clinician and by investigator (based on data from assesor), and only the added reference items from the assessor are considered in the Tx evaluation; c-A, continually added by clinician and subsequently by the investigator (based on data from assessor), and all added reference items are considered in the Tx-outcome; NA, no addition. n TDC ≤ −0.379 and n TDC > −0.379: number of patients for which TDC ≤ −0.379 or TDC > −0.379 respectively. n discrepancy rule: number of patients with application of the ‘discrepancy rule’ (see text, section ‘treatment procedure’). n S-Tx and n U-Tx: number of patients with a successful Tx and a unsuccessful Tx respectively. Note that n S-Tx = [(n TDC ≤ −0.379) – (n discrepancy rule)], and n U-Tx = [(n TDC > −0.379) + (n discrepancy rule)]. Note also that the application of the discrepancy rule was occasional, i.e. for 3.4-7.6% of the patients.

Success rate (%) = (n S-Tx/118)×100% (118 = total number of patients).

*, values based on n = 89 patients who entered the follow-up at EM according to separately added reference items (s-A) from the assessor (the mode of addition used in the RCT of the present study). Because the number of patients entering the follow-up at EM would have been slightly larger according to NA (n = 92) than actually occurring according to s-A (n = 89), the success-rate (n S-Tx) at LM might be slightly underestimated for the mode NA. The success-rate at LM is approximately correctly estimated for the mode c-A as the number of patients entering the follow-up was nearly the same for c-A (n = 88) as for s-A (n = 89). Note that regardless of a possible underestimation of n S-Tx for LM, NA, the value of success-rate is the smallest for LM, s-A.

Table 5 shows the mean and SD values of TDC following treatment, for the three modes of addition of reference items. These TDC-values are shown for the entire sample of patients (n = 118) and for the sub-sample of patients whose added reference items were at least based on data of the assessor; n = 33), for which the inter-mode differences were the largest. The top half of Table 5 shows the TDC-values when the initial Contrast-value of added reference items at the visit of addition was calculated with respect to the baseline according to equation (1). Since the score value of such an item had a high level of ‘3 ‘or ‘4’ at the visit of addition, and their scores at baseline were either ‘0’ or ‘1’, the initial Contrast-value had a positive value within a range from +0.5 (=(3 – 1)/(3 + 1) to +1 (=(3 – 0)/(3 + 0) or (4 – 0)/(4 + 0)). The bottom half of Table 5 shows the TDC-values when a value of zero was attributed as initial Contrast-value of added reference items. Whereas the use of positive initial Contrast-values enhances the sensitivity of TDC to detect cases of relapse of myogenous TMD, the use of initial zero values might be prefered in view of equal treatment of basic and added reference items (cf. Discussion). Significant inter-mode differences occurred in all cases (1-way-ANOVAs for repeated measures; p < 0.0001-0.05). With positive initial Contrast-values of the added items, the difference in TDC with respect to the mode non-addition became large for the subgroup of patients, up to 42% for the mode of separately added items (Table 5, top half). With zero values as initial Contrast-value of added items, the TDC-values of modes with addition of reference items approached closely those of the mode non-addition (Table 5, bottom half; even some non-significant differences in Bonferroni’s multiple comparison tests). The effect of using initial zero Contrast-values rather than positive Contrast-values was small and non-significant on the success rate in the long-term, i.e. 57.6% (68/118 patients) rather than 55.9% (66/118 patients) for the mode of separately added items.

**Table 5:** TDC-values for different modes of addition and initial Contrast-values of reference items

**Group**	**Occasion**	**Mode of addition**			**% difference relative to NA**		**Test**	**p-level**
		**s-A**	**c-A**	**NA**	**s-A**	**c-A**		
*use of positive value relative to baseline as initial Contrast-value for added reference items*								
all	EM	-0.635	-0.653	-0.659	3.6	1.0	s-A vs. NA	***
patients		(0.300)	(0.283)	(0.270)			c-A vs. NA	ns
n = 118							s-A vs. c-A	***
all	LM	-0.541	-0.556	-0.585	7.5	4.9	s-A vs. NA	****
patients		(0.339)	(0.324)	(0.298)			c-A vs. NA	*
n = 118							s-A vs. c-A	**
								
patients with	EM	-0.420	-0.454	-0.514	18.2	11.8	s-A vs. NA	****
added items		(0.333)	(0.309)	(0.276)			c-A vs. NA	**
from assessor							s-A vs. c-A	**
n = 33								
patients with	LM	-0.229	-0.268	-0.395	42.0	32.0	s-A vs. NA	****
added items		(0.281)	(0.271)	(0.254)			c-A vs. NA	***
from assessor							s-A vs. c-A	**
n = 33								
								
*use of zero as initial Contrast-value for added reference items*								
all	EM	-0.651	-0.665	-0.659	1.2	-1.0	s-A vs. NA	**
patients		(0.274)	(0.262)	(0.270)			c-A vs. NA	ns
n = 118							s-A vs. c-A	**
all	LM	-0.570	-0.583	-0.585	2.5	0.3	s-A vs. NA	***
patients		(0.303)	(0.293)	(0.298)			c-A vs. NA	ns
n = 118							s-A vs. c-A	*
								
patients with	EM	-0.485	-0.498	-0.514	5.6	3.1	s-A vs. NA	**
added items		(0.274)	(0.267)	(0.276)			c-A vs. NA	ns
from assessor							s-A vs. c-A	ns
n = 33								
patients with	LM	-0.342	-0.357	-0.395	13.3	9.5	s-A vs. NA	***
added items		(0.230)	(0.225)	(0.254)			c-A vs. NA	*
from assessor							s-A vs. c-A	ns
n = 33								

mean and (between brackets) SD values of TDC for two ways of attributing an initial Contrast-value of an added reference item at the visit of addition, i.e. (i) by calculating the Contrast between the large score value at addition with respect to the low value at baseline using equation (1) (see text), or (ii) by attributing the value zero as initial Contrast-value (cf. Discussion). For explanation of the abbreviations under ‘occasion’ and ‘mode of addition’, see Table 4. Test: Bonferroni’s multiple comparison test which could always be applied as all 1-way ANOVAs for repeated measures were significant (p < 0.0001-0.05). p-level: ****, p < 0.0001; ***, p < 0.001; **, p < 0.01; *, p < 0.05; ns, non-significant.

### Control on regression to the mean

**Figure 4:**
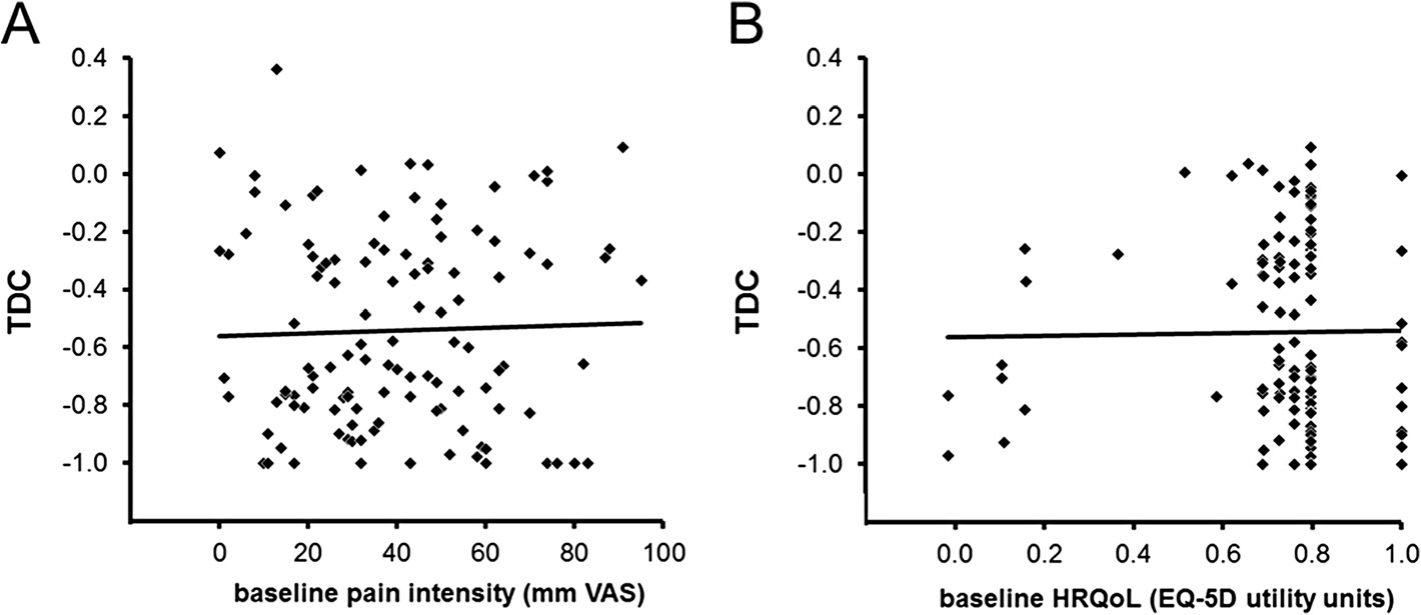
**Relationships between TDC-values and baseline values of intensity of predominant pain from the masticatory system (A) and general Health-related Quality of Life (B).** These baseline values are related to the severity of myogenous Temporomandibular Disorders in individual patients; the values of HRQoL in an inversely way. The TDC-values are from the last evaluation visit at LM (last measurement) following treatment and include possibly a separate addition of reference items by clinician and investigator (based on data from assessor) and considering only the added items from the assessor. For TDC = 0, an overall change in signs and symptoms is lacking following treatment and for TDC = −1 all signs and symptoms of myogenous TMD have disappeared. Solid lines, regression lines: TDC = 0.00047.PI – 0.560, in which PI is pain intensity (Pearson’s correlation coefficient: r = 0.031, not significant, n = 118), and TDC = 0.0245.HRQoL-0.564, in which HRQoL is Health-related Quality of Life (r = 0.015, not significant, n = 112, 6 missing values). Similarly, no significant relationships were observed for TDC from the end measurement (EM) following treatment in the short-term and for other modes of addition of items, including no-addition. Note that significant regressions are lacking while the scatter in TDC-values is similar within the range of baseline values, indicating that (i) the TDC-values from individual patients are independent from their baseline values of pain intensity or HRQoL, and (ii) a similar variety of relative change following treatment occurs for the various patients, regardless of the baseline severity of myogenous TMD. The fraction of patients whose TDC-value has dropped to or beyond the cut-off level of −0.379 and thus the chance of attaining functional status, is independent from the patient’s baseline severity level.

A highly significant (p < 0.001) regression occurred between the raw difference values in VAS-scores of pain intensity between, for example the last measurement (LM) and baseline, and the baseline values (r = 0.60, n = 118) of the various patients. This significant regression, with a negative gradient, was due, at least in part, to regression to the mean. In agreement with mathematical considerations (Appendix, section ‘Lack of bias by regression to the mean in Contrast and TDC-values’), any regression was lacking (r = 0.038) between the Contrast-values of pain intensity at LM (ratio between difference and sum of scores at LM and at baseline) and the baseline scores of pain intensity. Any regression was also lacking in relationships between TDC and the baseline of a variable that is related to severity of myogenous TMD in individual patients. Thus the TDC-values in the short-term following treatment (at EM) or in the long-term (at LM) did not depend on the level of the predominant pain at baseline (Figure 4A). Pearson’s correlation coefficient of these regressions was nearly zero (r = 0.013-0.066), whether or not reference items had been added during treatment and/or follow-up. Furthermore, the scatter of the TDC-values was similar within the entire range of baseline values of pain intensity. The TDC-values were also independent from the baseline utility values of Health-related Quality of Life (Figure 4B), a variable which is to some extent inversely related to severity of the myogenous TMD.

### Validation of the cut-off point of TDC for deciding successful treatment

The present study provided three ways of validation. First, it is of interest to consider the distribution of the TDC-values which became bimodal in the long-term, at the last measurement (LM; Figure 3B). The first peak (pronounced negative TDC-values) in this bimodal distribution corresponded to a great extent to patients whose treatments were successful according to the criterion of TDC ≤ −0.379. The second peak corresponded to a great extent to patients with an unsuccessful treatment (TDC > −0.379).

**Figure 5:**
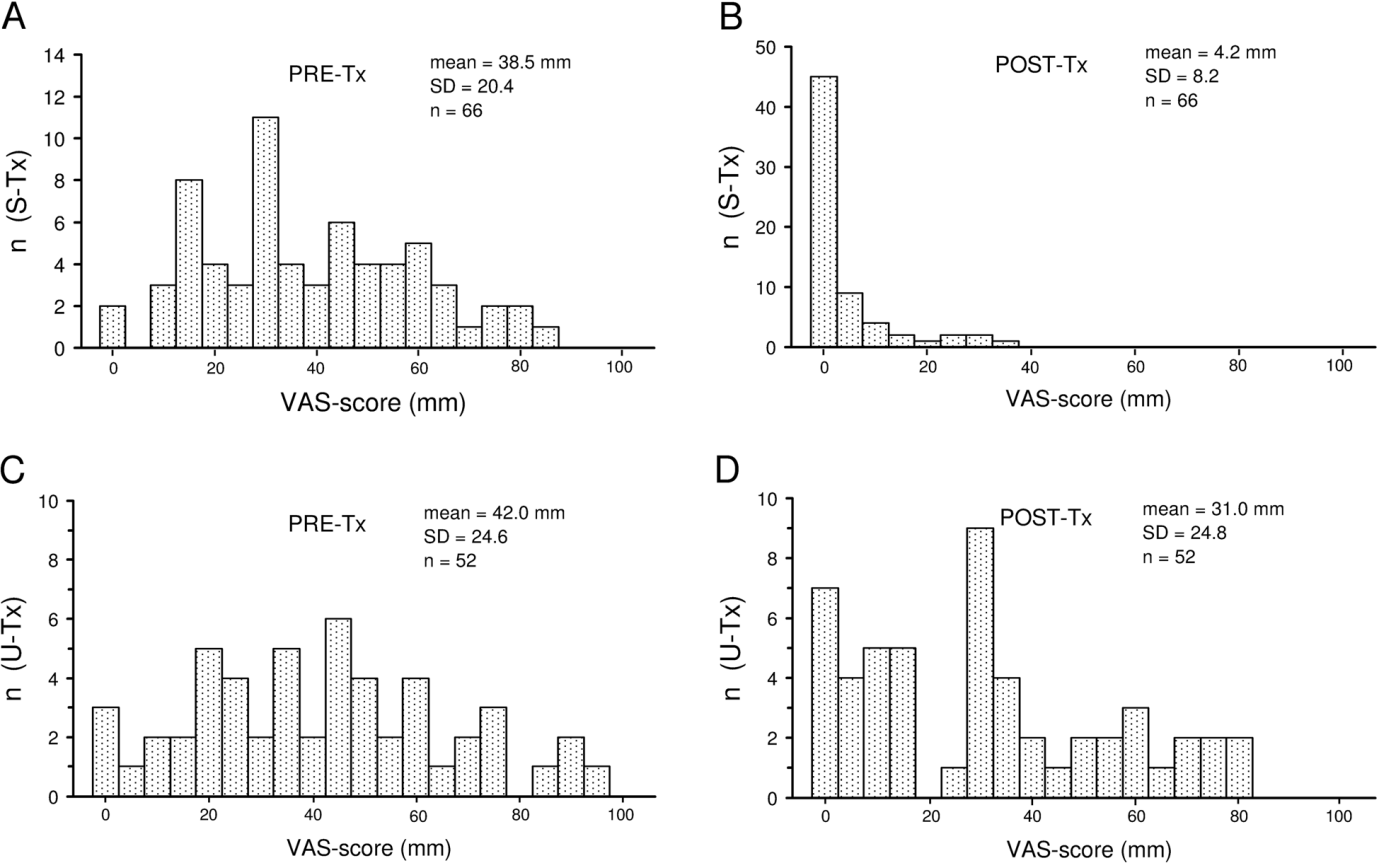
**Distributions of VAS-scores of the intensity of predominant pain from the masticatory system.** n, number of patients. S-Tx and U-Tx, patients with a successful treatment in the long-term **(A-B)**, and an unsuccessful treatment **(C-D)**, according to TDC based on data from the assersor and an occasional use (3.4%) of the discrepancy rule. Pre-Tx, pre-treatment VAS-scores **(A,C)**; Post-Tx, post-treatment VAS-scores **(B,D)** from the last evaluation visit at LM (‘last measurement’).

Second, also for comparing treatment effect of the TDC-procedure with that of other procedures (cf. Discussion), it is of interest to examine raw changes in pain intensity in a traditional manner. To that end, the distributions of the values of intensity of the predominant pain (VAS-scores) were considered before and after treatment. The intensity of the predominant pain in the oral system is a key outcome variable as it is related to function impairment of the patients suffering from myogenous TMD. The wide pre-treatment distribution of VAS-scores of pain intensity (Figure 5) only changed into a narrow distribution of small post-treatment VAS-scores (p < 0.01; Wilcoxon’s test for paired observations), for patients whose treatment was successful at LM, using TDC. The pre-treatment distribution did hardly change for patients whose treatment was unsuccessful according to TDC (Figure 5). The percentage decrease in VAS-score was 90.5% (SD 16.5; n = 66), when averaged across the various patients whose treatment was successful.

Third, Health-related Quality of Life (HRQoL) increased significantly (p < 0.0001; Wilcoxon’s test for paired observations) from 0.728 (SD 0.234) to 0.916 utility units of EQ-5D (SD 0.143, n = 63, 3 missing pairs) for those patients whose treatment was successful at LM, using TDC. HRQoL did not change significantly from 0.734 (pre-treatment; SD 0.129) to 0.662 units (post-treatment, LM; SD 0.287, n = 48, 4 missing pairs) for patients whose treatment was unsuccessful.

## Discussion

### Characteristics of the TDC-procedure

The current TDC-procedure includes a set of rules, i.e. (i) those regarding adaptive item selection before, during treatment and separately at the post-treatment occasions of evaluation, (ii) the rule based on relative decrease in scores for progressing or ending treatment, and (iii) the discrepancy rule in which the patient’s demand for subsequent treatment can overrule the conclusions of the clinical examination. This TDC-procedure approaches clinical care of myogenous TMD to such an extent that the clinicians who participated in the Randomized Controlled Trial of the present study, felt confident to use TDC for deciding when to end treatment in a standardized manner. Despite the abovementioned rules which may influence treatment outcomes in specific ways, it is still possible to compare treatment effect between the current TDC-procedure with that of other procedures, even ones which differ considerably, i.e. a traditional Routine Outcome Monitoring or a traditional Randomized Controlled Trial. To that end, it is of interest to analyze some key outcome variables in a traditional manner. Two parameters of raw change in outcome variables are of interest in this respect, i.e. Clinically Important Difference (CID) and Cohen’s Effect Size (ES). CID is the mean raw change in an outcome variable observed in a patient sample after interventions of known efficacy [20]. The ratio between the mean change following a therapy and the SD of the baseline scores is ES for this therapy [21,22]. A value of ES of 0.2 or less represents a small change, a size of 0.5 represents a moderate large change and a value of 0.8 or larger corresponds with a large effect of therapy. Apart from comparing different therapies within the same procedure, for example a traditional Randomized Controlled Trial, CID and ES can also be used to compare the effect of different procedures using the same type of treatment. CID with its mean and SD values is then suitable for statistical testing of efficacy between different procedures. When CID and ES are applied to an entire patient sample, these parameters refer to an overall procedure effect, regardless of how the patients are divided in a procedure-specific way into two groups, with a ‘successful’ and an ‘unsuccessful’ treatment.

The effect of the current TDC-procedure is large for myogenous TMD, i.e. Cohen’s effect size (ES) is 1.09 and 1.38 for rating of pain behaviour or pain intensity respectively [18]. Thus the current TDC-procedure has proven to be effective for patients who were, like in a traditional Randomized Controlled Trial, selected using stringent criteria (Appendix, ‘Inclusion and exclusion criteria of the patients’ , [17]). ES observed in myogenous TMD patients is similar to an ES of 0.80 and 1.38 ([23], based on disability due to pain) for patients receiving physical therapy for low back pain [24] or acute shoulder pain [25] respectively.

TDC deals with multiplication factors of relative change in score values rather than with these values themselves. TDC is therefore not bound to a particular scale, and arbitrary weighing of score values from different items is avoided (cf. Appendix, section ‘averaging of ratio values between scores from two times of measurement’). On the other hand, the multiplication factors related to relative change of different items have been equally weighted. The rationale of this equal weighing was that all items were related to intensity or frequency pain from the masticatory system, and to disability of this system due to the presence of pain. Furthermore, equal weighing is supported by the finding that the expectation of patients with facial pain or fibromyalgia regarding treatment of their symptoms is constant in a relative sense for several domains of scoring [14].

Apart from a 100 mm VAS for intensity of predominant pain, adjectival 0–4 point scales (giving a choice between 5 states) have been used for all other items to reduce the time-load of patient and clinician. Such scales are sufficiently graded for myogenous TMD as the mean treatment effect is large for this disorder, i.e. Cohen’s effect size is 1.09-1.38 (see above). The accuracy gain of more detailed scales is limited because subjects are mentally able to handle only five to nine levels and will thus mentally reduce more detailed scales to about seven segments [26,27]. The discrete score values are on an ordinal rather than an interval or ratio scale level. However, as the underlying phenomenon (disorder activity) is on an interval scale, these measures can be analyzed parametrically if the sample size is large enough (central limit theorem). Because TDC is a mean of several Contrast-values, the gradation of TDC-values is larger than that of Contrast-values.

The influence of random fluctuations on TDC is limited by selecting items for an adaptive way of testing, using values of the smallest detectable difference (SDD) for single scores (2–3 units) as a threshold. This selection does not introduce a risk on introducing bias by regression to the mean in the TDC-values. Time effects by chance are neutralized when the criterion for a successful treatment is based on a constant amount of relative change rather than on criteria which are related to raw change. Regression is lacking in the relationship between the Contrast-value of a single variable and its baseline and the bandwidth of scatter in the Contrast-values is constant (cf. Appendix, section ‘Lack of bias by regression to the mean in Contrast and TDC-values’). Hence, Contrast-values of any item that contributes to a TDC-value are independent from their baseline values. As each of the items which are involved in the mean Contrast-value (hence the TDC-value) is related to the baseline level of severity of myogenous TMD, a regression will also be lacking in the relationship between TDC and baseline values of items like predominant pain of the masticatory system (VAS-scores) and Health-related Quality of Life (EQ-5D utility units). A lack of such a regression has been observed indeed (Figures 4 A-B). The constant bandwidth of scatter of the post-treatment TDC-values around the nearly horizontal regression line means that a similar variety of TDC-values (similar variety of relative change) from different patients occurs, regardless of the severity level of the patients’ myogenous TMD. Hence, the fraction of patients whose TDC-value has dropped to or beyond the cut-off level of −0.379 and thus the chance of attaining functional status, are independent from the patient’s baseline severity level.

Also related to selecting items in an adaptive way, one might question whether statistically, an overall reliable change (RC > SDD for score means) can be achieved in patients with a low baseline, who have only reference items of ‘2’ of which some scores decrease by merely one unit rather than consistently by two units. This problem has been avoided in the present study by requiring a low general score level (reflected as TDC ≤ −0.379) during subsequent occasions of treatment evaluation rather than at one occasion in the traditional concept of RC related to SDD. A patient’s general score level had to be low at the last two occasions of the clinician’s evaluation before treatment was considered as being potentially successful. Subsequently, the general score level had to be low at three successive post-treatment times of the assessor’s evaluation (5 weeks, 6 and 12 months after treatment), before treatment was ultimately considered as being successful. A repeated end evaluation will also correct a single decision of a ‘successful’ treatment which might be false due to intra-subject variation.

The current sample of 118 patients included 2 patients whose general level of baseline scores was somewhat higher than that of the patient from the pilot sample whose general baseline level was used for tuning the cut-off value TDC = −0.379. The mean score level of these 2 patients, averaged across the reference items, was only slightly larger than the one of the patient used for tuning the cut-off point, because the mean level was largely dominated by many maximal score values of 4 units (cf. Appendix, section ‘choosing two cut-off points of TDC’). The TDC-value required to attain the zone of functional status for these patients was therefore only slightly more negative than TDC = −0.379, hence the required treatment factor *T* was only slightly larger. One might argue that the decision of a successful treatment of these 2 patients might have been favoured by the use of a slightly less conservative cut-off value TDC = −0.379. However, the criterion TDC ≤ −0.379 for a successful treatment has been used in combination with the discrepancy rule in which the patient’s demand for subsequent treatment can overrule the conclusions of the clinical examination. Such a combined use is actually more effective than a more negative TDC-value as cut-off. Apart from enabling further treatment for patients with such a demand, the combined use prevents over-treatment of some patients who would have been classified as being unsuccessfully treated using a more conservative cut-off value of TDC while these patients had no demand for further treatment in the current procedure (cf. section ‘validation’ below). Thus the TDC-procedure has been proven to be robust.

The baseline data of a traditional Routine Outcome Monitoring (ROM) include all scores regardless of their level. In accordance with common clinical care, large scores related to items of late pronounced signs or symptoms are automatically included in the overall outcome variable of such ROM and might influence treatment duration, success rate and efficacy of a therapy. Such large scores might also be included in the outcome variable of a traditional Randomized Controlled Trial (RCT) of which the treatment duration is constant, and influence success rate and therapy efficacy. While the influence of scores which are temporarily large during the treatment will wane in the outcome variable of a traditional ROM or RCT, only scores that are sustained large beyond the end of treatment, and scores which become large during a follow-up will influence the post-treatment outcome variable related to these procedures.

The baseline scores of the TDC-procedure are adaptively selected for being sufficiently pronounced. Reference items related to late pronounced signs and symptoms must be added later during treatment and follow-up to comply with common clinical care to follow any item with a high level of severity and serving the safety of patients who participate in a randomized controlled trial. The overall effect of added reference items is small in the present study because even in the fraction of patients (44.1%) in which addition of reference items occurred, there were much more basic reference items involved (84%) than added reference items (16%). Furthermore, addition of reference items was, in general, concomitant with increased score levels of basic reference items. Such an increase in the general severity level of the patient’s myogenous TMD is reflected in mean levels of post-treatment TDC that, regardless of the mode of addition, are clearly larger (less negative, indicating less improvement) for a subgroup of patients with added reference items, than for the entire patient sample (Table 5). Addition of reference items therefore occurred more frequently in patients whose treatment was unsuccessful, for example, in 84% of such patients at the last post-treatment measurement. Thus, even in patients with added items, relative changes in the basic reference items dominate the outcome.

The increase in severity level of myogenous TMD, to which the addition of reference items during treatment is related, will extent the duration of treatment even when this increase is temporarily. More visits are then required before a clinician can decide, using the TDC-criterion (TDC ≤ −0.379), that a treatment has become potentially successful.

As the addition of reference items occurred only in patients who had a moderately large number of basic reference items at most, their general score level remained below that of the TMD patient with maximal baseline values from the pilot sample to which the treatment factor *T* was tuned *a priori*. Thus the criterion of reaching the zone of functional status following an overall decrease in score values by at least the treatment factor *T* (reflected as TDC ≤ −0.379), remains valid for patients with added items.

In the current Randomized Controlled Trial with TDC, the initial Contrast-value of an added reference item was determined with respect to its basic value using equation (1). Since the score value of such an item was either ‘3’ or ‘4’ at the visit of addition, and their baseline score values were either ‘0’ or ‘1’ , the initial Contrast-value varied within a range from +0.5 to +1.0, where the positive sign reflects a worsening with respect to baseline. Attributing such a positive Contrast-value will enhance the sensitivity of TDC to detect cases of relapse as this Contrast must be compensated by negative Contrast-values from several other items (reflecting improvement for these items) before the criterion TDC ≤ −0.379 might be attained for a successful treatment. The positive Contrast-value will decrease to zero when the patient does not improve for that item, and will have a negative value when the patient has improved at a visit following the visit of addition. Thus even if the initial positive Contrast-value of an added reference item is decisive for non-attaining functional status, a decrease to zero or to a negative value during a subsequent visit will enhance the chance on attaining functional status at a later phase of treatment.

In contrast to an addition before the end of treatment, the initial positive Contrast-values of reference items that are added at the last visit of treatment or at the one of the post-treatment occasions will have a relatively large weight in the parameters of treatment outcome (success rate and treatment efficacy) at the end of treatment or following treatment respectively. If the initial positive Contrast-value is decisive for a post-treatment outcome of a non-successful treatment, a subsequent visit for improvement is lacking because a further follow-up was ended as soon as a patient’s treatment was considered as being unsuccessful at one of the three occasions of post-treatment evaluation. Although not significant, the success rate therefore tends to be lower for the mode of separately added items in which only added items based on data from the assessor are considered with respect to the mode of continued added items from clinician and assessor, or to the mode of non-addition (Table 4). Furthermore, the post-treatment values of TDC (treatment efficacy) are significantly larger (less negative; less improvement) for the mode of separately added items.

In accordance with clinical care, the initial treatment of myogenous TMD patients, suffering from chronic pain, was stepped up or changed for ethical reasons, when this treatment (which could have a duration within a range from 6 to 30 weeks) appeared to be unsuccessful at a post-treatment occasion of evaluation. Hence, a waiting period was not applied for these patients to complete the follow-up of a year during which spontaneous improvement might have occurred in some patients. Thus regardless of the procedure used for treatment evaluation, including the TDC-procedure, not completing the follow-up of all patients will yield some bias in success rate and therapy efficacy, i.e. both parameters will probably be slightly underestimated.

Once an item has been added as a reference item in the TDC-procedure its Contrast-values remain to contribute to the TDC-values of subsequent visits, also if the score value of that item wanes to zero (its Contrast-value becomes then −1). When the same item is detected as an added reference item during treatment as well as at the first post-treatment visit, its Contrast-value at the first post-treatment visit might differ between the modes of separately added reference items from clinician and assessor and continued added items respectively. This inter-mode difference occurs in particular when the initial Contrast-value of the added item is determined with respect to the baseline. For example, suppose that the clinician observes a score value of ‘4’ at a treatment visit of an item of which the baseline is ‘0’ , the initial Contrast-value is then +1 (=(4 – 0)/(4 + 0) at the treatment visit of addition. Suppose that the assessor also observes a score value of ‘4’ at the first post-treatment visit. If the treatment period is considered as a black box (as in the mode of separately addition of reference items), the initial Contrast-value with respect to baseline is again +1 at the post-treatment visit. On the other hand, the Contrast-value is not +1 but zero in the mode of continued added items (no separation of item information between treatment and post-treatment period), with respect to the score value at the visit of addition during treatment (=(4 – 4)/(4 + 4)). This zero value reflects no change in score value between the post-treatment visit and the treatment visit of addition. Thus the possible difference in post-treatment Contrast-values of added reference items yields some inter-mode bias in the post-treatment TDC-values, which has, however only a small and non-significant effect on success rate (Table 4). Success rate is hardly affected because attaining or passing the TDC-level of −0.379 is decisive for considering treatment as being potentially successful rather than the TDC-value itself. Thus possible inter-mode variations are irrelevant in view of success rate, for TDC-values which are sufficiently remote from the cut-off level of −0.379. Although small in the entire patient sample (<3%, Table 5, top), the inter-mode bias has a significant effect on the post-treatment TDC-values as a measure of therapy efficacy.

Using the mode of separately added reference items makes the post-treatment TDC-related outcome variables free from any possible clinician-bound bias. One might argue that a clinician-bound bias would also be avoided when the anamnestic and clinical examinations of a patient would solely be carried out by a blinded assessor, also at the treatment visits. The investigator or a computer system could transfer the score and TDC information to the clinician to keep the assessor blinded. A continued mode of addition of reference items could then be applied while avoiding a possible clinician-bound bias as well as an inter-mode bias of addition. However, for two reasons, even an improved mode of continued addition is not appropriate for a Randomized Controlled Trial which uses TDC and allows variation in the number of visits and the duration of treatment.

First, apart from a more time consuming thus less feasible procedure for the assessor there might be a risk on a less natural interaction between clinician and patient which might influence treatment outcome. While this risk might be present for chronic pain patients whose data of evaluation originates in part from manual clinical tests, such a risk is absent when exclusively questionnaires are used of which the data are collected by a person who is neither a clinician nor an assessor, like in Routine Outcome Monitoring of psychiatric patients [11-13].

Second, the chance of detecting added reference items with large score values, transient ones in particular, may depend on the frequency of visits and the duration of treatment. For example, suppose that a sign or symptom, which is related to a potential added reference item, reaches a high level for a short time. The likelihood of detecting the high score level of such an item is then larger with a higher frequency of visits. Furthermore, suppose that the scores of more items are transiently increased. With a particular frequency of visits, detection of one of these items will then occasionally occur when the timing of a high score level coincides with that of a measurement. Such a coincidence will occur more likely with a longer duration of treatment. The therapies used in the current Randomized Controlled Trial differed in a therapy- and patient-specific way in number of visits and in treatment duration. Although neither the frequency of addition nor the number of added items differed significantly between therapies at the end of treatment, application of the mode of separately added reference items from clinician and assessor, is *a sine qua none*. Thus the treatment period was considered as a black box in the present study for determining the post-treatment TDC values, to avoid any bias which might be due to variation in number or frequency of visits and duration of treatment. Furthermore, the number of post-treatment visits for treatment evaluation by the assessor and their intervals were the same for the various therapies. As explained before, the mode of separately added reference items yields an inter-mode bias of the post-treatment TDC-values when the baseline of an added reference item is used for determining its initial Contrast-value. Below it will be shown that by using zero as an initial Contrast-value, which can be theoretically expected, the inter-mode bias is largely diminished.

Basic reference items and added ones have been unequally treated in the current TDC-procedure with respect to the reference score value used for determining Contrast-values. For basic reference items, the Contrast-values have always been determined with respect to the same reference score values from the visit at which the items were detected as reference items, i.e. the values for the pre-treatment ‘visit’. In contrast, two reference values have been used for an added reference item, i.e. (1) the pre-treatment score value for the Contrast-value at the visit of addition and (2) the score value from the visit of addition (visit of detection) for Contrast-values at subsequent visits (the second situation is equivalent to that of basic reference items). The use of a reference score value can be confined to the same value from the visit of addition by considering which Contrast-value should be applied within the visit of addition, from a theoretical point of view. A patient has a particular pattern of levels of signs and symptoms at a particular moment within a visit. This level pattern is related to an intrinsic score pattern which becomes known following measurement. It is then also known which items will become added reference items. Measurement, for example, scoring of pain intensity during palpation of a sore jaw muscle can only be carried out once within a visit because the outcome of a second measurement will be influenced by the first one. However, even without a second measurement, it is known that the intrinsic score pattern of a second moment will be identical to the first intrinsic pattern if the interval between the two moments is infinitely small. Hence, with no change in the intrinsic score values, the Contrast-value of any added reference item will be zero between the second and the first moment. A zero Contrast-value could also be attributed to basic reference items at the pre-treatment ‘visit’ of their detection. Thus all reference items, basic ones as well as added ones, are treated equally regarding the use of their reference values, if the initial Contrast-value at the reference visit is set to zero.

The post-treatment TDC-values are clearly less negative for the mode of separately added reference items than for the mode of continued added items or the mode of no-addition, when the initial Contrast-value has a positive value, i.e. the one with respect to the baseline (Table 5, top). When initially zero Contrast-values are used for added reference items, the difference in the post-treatment TDC-values becomes marginal between the modes of separately added items and the mode of continued added items (2.2% for the entire sample, <4% for a sub-sample with added items, Table 5, bottom). Thus the inter-mode bias in the TDC-values which occurs when the Contrast-values of added reference items have initially a positive value, is largely eliminated by the use of initial zero Contrast-values. The TDC-values with added items approach then even closely the TDC-values without added items. The success rate at the last post-treatment visit becomes also slightly less conservative by using initially zero Contrast-values, i.e. 57.6% (68/118 patients) rather than 55.9% (66/118 patients) for the mode of separately added items.

Hence, the use of initially zero Contrast-values for added reference items is recommended in future studies. A TDC-procedure which is further similar to the one from the present study, including using the mode of separately added reference items allows then monitoring of late pronounced signs and symptoms during treatment, and will yield objective data on the number of visits needed for treatment and treatment duration. The data on visits and treatment duration would even be free from any possible clinician-bound bias if it is possible or feasible that the data for evaluation at the various visits of treatment are obtained by a non-clinician (it remains then essential to consider the treatment period as a black box regarding the post-treatment TDC-values). Based on the TDC-data from a blinded assessor, a procedure with separately added reference items, including the use of initially zero Contrast-values, will yield nearly unbiased data on success rate and efficacy of treatment.

In the present study, scores were used that have a zero value when there is no pain or impairment and a maximal value when the extent of pain or impairment is greatest. The Appendix (section ‘The use of TDC on scales with a reversed meaning’), outlines how to handle scales with a reversed meaning. Furthermore, the Appendix (section ‘The use of TDC when *a priori* knowledge of an item’s unimpaired value is lacking’) outlines how to use TDC when the value of a score corresponding to ‘least impairment’ is *a priori* unknown for a patient.

### Differences between TDC and ROM procedures

A TDC-procedure differs in three aspects from a traditional Routine Outcome Monitoring (ROM). First, in the TDC-procedure, items with sufficiently large score values either at baseline or during treatment are selected as reference items for monitoring relative change. Thus relative change is tested adaptively only for those signs and symptoms which are statistically pronounced and are of interest for the clinician to follow. By contrast, all items of a multidimensional questionnaire are included in a traditional ROM using raw change. Apart from a possible difference in sensitivity to detect change between a TDC-procedure and ROM, the outcome value of ROM might be more ambiguous than that of a TDC-procedure. If large score values of items would wane during treatment and would be replaced by large scores values of other items, such an event will not be reflected in the summed or averaged outcome variable of ROM. A ROM outcome at a particular visit only reflects a mean actual state. In a TDC-procedure, the detection of items with sufficiently pronounced scores is always concomitant with the attribution of a reference score value which is used to determine a Contrast-value. Once an item has been detected as a reference item, its Contrast-values contribute to the TDC-values of subsequent visits. Thus TDC has a ‘memory’ which requires (as explained above) to consider the treatment period as a black box for obtaining unbiased outcome variables in a Randomized Controlled Trial with variable treatment duration.

Second, the TDC-procedure differs from the traditional Routine Outcome Monitoring (ROM) in the end level of the scores and also likely in treatment duration. Using TDC, the smaller a patient’s baseline is, the end scores following successful treatment will be closer to zero, hence more remote from the Upper Limit of Functional Status (*ULFS*; Figure 1). In order to decide that a treatment is successful, two criteria are used in a traditional ROM, i.e. (1) a decrease in averaged score values from a questionnaire should exceed the Smallest Detectable Difference (SDD) for such averaged scores, and (2) the end level of the averaged scores should have passed *ULFS*. In Contrast to the TDC-procedure, the end levels will therefore tend to be closer to *ULFS* in ROM. Treatments of patients whose baseline is located just above the *ULFS* at a small distance of SDD for a score average, and whose end score drops just below *ULFS*, will then be considered as being successful. If such a high end level occurred in chronic pain patients by using a ROM based on raw change while the perception of improvement by treatment is associated with relative change [15], there might occur a discrepancy between a favourable ROM outcome and a patient’s perception of only a small improvement (cf. Background). Such a discrepancy might increase the risk on relapse. End levels as controlled by TDC that are proportional to the baseline concur with a relationship between relative decrease in pain intensity and the patient’s assessment of treatment effect that is independent from the baseline in chronic pain patients [15]. If treatment success is associated with the patient’s assessment of treatment effect of, for example, ‘much improved’ or better, then this assessment is related to a particular relative decrease in pain intensity. Such a decrease, applied as a multiplication factor to a patient’s baseline of pain intensity will yield an end level of pain intensity that is proportional to the baseline (Figure 1). Further research is required to examine the extent to which a relationship between relative decrease in signs and symptoms and assessment of treatment effect occurs in general in diseases and disorders and whether the risk on relapse will be smaller with TDC than with procedures using raw change.

A third difference between the TDC-procedure and a traditional ROM concerns regression to the mean. Whereas bias by regression to the mean does not occur with TDC-values as explained before, a raw change in score level, for example the change in score of pain intensity, will always show some regression to the mean of pain intensity or to the mean of any other variable that is related to severity of the disorder. ROMs using raw changes in score levels are thus susceptible to this artefact by which treatment effect might be somewhat overestimated, in particular when the threshold of signs and symptoms is chosen relatively high at the intake of the patients.

### Validation

The criterion of TDC ≤ −0.379 (in combination with the occasional use of the discrepancy rule) for distinguishing between a successful/unsuccessful treatment, has proven to be reliable on five grounds.

First, the distribution of the TDC-values is bimodal in the long-term, representing two groups of patients in respect of their treatment outcomes. The separation between the two groups might have been better still had a slightly more negative cut-off point been used, notably TDC ≤ −0.560 instead of ≤ −0.379 (Figures 3B, C-D).

The conclusion of a more negative cut-off for TDC is reinforced by another finding from the present study. The distribution of post-treatment scores of patients from the current large sample, whose treatment is successful, suggests that the level of the Upper Limit of Functional Status (*ULFS*) might be 1.08 units rather than 1.40 units as assessed by a panel using scores from a pilot sample (see Appendix, section ‘Choosing two cut-off points of TDC’). Such a lower level of *ULFS* would correspond to a more negative cut-off point TDC = −0.482 instead of −0.379. A limitation of the present study is that normative score values from Community Control subjects (CoCos) are lacking for the various items. Data from CoCos might improve the assessment of the *ULFS* and hence the determination of the cut-off value of TDC.

However, the use of a slightly less conservative cut-off value TDC = −0.379 in combination with the discrepancy rule in which the patient’s demand for subsequent treatment can overrule the conclusions of the clinical examination, is actually more effective than a more negative TDC-value as cut-off. If the criterion TDC ≤ −0.379 alone were used, 4 patients out of 118 (3.4%, Figure 3B) would have been classified as being successfully treated despite the fact that these patients disagreed with this conclusion. This disagreement was solved by application of the discrepancy rule. Using a more negative TDC-value (−0.560), 2 of these 4 patients would have been correctly classified as having had unsuccessful treatments. Three other patients, however, would then have been classified as being unsuccessfully treated whereas these patients had no demand for further treatment. Thus the use of a less conservative cut-off TDC-value in combination with the discrepancy rule has prevented over-treatment of these three patients.

The finding that TDC-anamnestic (patient assessing daily functioning) indicates less improvement than TDC-clinical from the clinician, supports the use of the discrepancy rule. TDC-anamnestic remained constant from the end of treatment while TDC-clinical increased when another person than the clinician carried out clinical testing. The more favourable clinician’s value for TDC-clinical might, apart from a clinician-bound bias, be related to a patient’s tolerance to clinical testing that develops during treatment and is clinician-bound.

A second ground of validity is that the VAS-scores of pain intensity become residual in the long-term in patients whose treatments were successful according the TDC-criterion (Figure 4). Thus 55.1% of the VAS-scores is ultimately zero, and even the maximal value (35 mm) is smaller than the long-term SDD of VAS-scoring (49 mm [18]). Being a reference item of TDC, this decrease in VAS-score has occurred with a simultaneous decrease in score values of other items.

A third ground of validity is that the increased utility values of EQ-5D (a variable that is independent from TDC) in patients whose treatment is successful (mean 0.917 units), corresponds to a self-rated global health of a general population sample that is ‘good’ to ‘very good’ [28].

A fourth reason for validity would have been provided in a direct manner by an association between relative decrease in the score of pain intensity and the assessment of treatment effect by the various myogenous TMD patients. However, a limitation of the present study is that assessment scorings are lacking. A second-best solution is considering the association between mean percentage decrease in score of pain intensity and the assessment of treatment effect by chronic pain patients, regardless of their baseline of pain intensity (Figure eight in reference [15]). The mean decrease of 90% that occurred in the TMD patients with a successful treatment according to the TDC-criterion, is related to ‘very much improved’ or better in the perspective of chronic pain patients. A decrease of 55% in pain intensity that corresponds to the cut-off point TDC = −0.379 is related to ‘much improved’ or better. The cut-off point TDC = −0.212 (decrease of 35% in pain intensity) used to distinguish between sufficient/insufficient responsiveness is related to ‘moderately improved’ (between the categories ‘minimally improved’ and ‘much improved’).

A decrease of 55% in pain intensity that corresponds to the cut-off point TDC = −0.379 corresponds also closely to the expectation of patients with facial pain or fibromyalgia regarding treatment of their symptoms [14]. The expected reduction of pain, fatigue, distress or interference with daily activities varied within a small range from 56% to 63%, regardless of the type of chronic pain patient.

A fifth ground of validity is that the success rate of treatment in the present study (75% in the short-term) is similar to that (75-80%) reported in TMD textbooks [29-31].

### Application

The purpose of TDC is to allow a clinician to make decisions on the progress and ending of a treatment in a standardized way that complies with clinical care to enable randomized controlled trials under natural conditions. As TDC enables an objective determination of treatment duration and the number of visits required in individuals, TDC will facilitate a costs-effectiveness-analysis of therapies. Like in a traditional Randomized Controlled Trial, stringent inclusion and exclusion criteria were applied to select the patient sample of the present study [Appendix, ‘Inclusion and exclusion criteria of the patients’, [17]. In particular, patients were excluded who had any previous TMD treatment (either a dental one or physiotherapy), or other treatments for pain (also nonfacial pain) more recent than a year. However, the TDC-procedure enables to determine treatment outcomes in future studies, while approaching more the usual patient intake of clinical care.

Apart from patients suffering from chronic pain and tenderness of the muscles of mastication, the TDC-procedure is potentially suitable for monitoring other disorders or diseases in which, as for chronic pain patients in general, the patients’ assessment of treatment effect is related to relative decrease in signs and symptoms, regardless of the baseline. A TDC-procedure might be of interest for psoriasis, a systemic chronic-inflammatory disorder affecting predominantly the skin. A primary outcome uses relative decrease in the Psoriasis Area and Severity Index (PASI) [32,33]. Correlations between decrease in PASI and the Dermatology Life Quality Index (DLQI) or a VAS-score of the patient’s assessment of psoriasis activity respectively are enhanced by considering relative change [34].

Using scores of key items from appropriate instruments, definition is only required of (i) a disorder-specific upper limit of functional status, and (ii) a disorder-specific cut-off point of TDC, which is related to a constant factor of relative change needed to pass this upper limit in patients with the largest levels of severity in the sample so that the cut-off point of TDC will be applicable to any patient. The upper limit of functional status is at a low level for myogenous TMD confining a zone of functional status of healthy people. This limit will probably be at a higher level for a degenerative disease or disorder because of a lack of potential of therapy to diminish signs and symptoms completely. For a patient with maximal scores, the degree of relative change required to attain a higher upper level of functional status will then be smaller than for myogenous TMD. Hence the cut-off value of TDC will then be larger (less negative) than −0.379.

Apart from the use as an index with a cut-off point to control treatment duration, this cut-off point of TDC is further used to classify treatment outcome dichotomously as being successful/unsuccessful, using data from a blinded assessor. Success rate between therapies can then be differentiated using non-parametric chi-square statistics.

Traditional single outcome variables may still be useful in powerful parametric statistical tests on unpaired observations to detect differences in efficacy between therapies, using mean and SD values. Post-treatment TDC-values might be less suitable for such tests as TDC is obtained by averaging Contrast-values with patient-specific numbers. The variance of TDC-values will therefore differ between patients. Traditional multi-dimensional scales in which raw score values are summed or averaged (used in traditional Routine Outcome Measurement) might have a similar variance problem as the scorings from all items contribute to the overall outcome value, including those items for which impairment is lacking. Patients with relatively small signs and symptoms might have a small overall outcome value with a small variance because of a large contribution of zero score values for which variance is lacking. Avoiding this floor effect by raising the intake threshold of the patients’ signs and symptoms will, in a traditional ROM but not in a TDC-procedure, increase the risk on confounding the treatment outcome with effects of regression to the mean. The variance problem might be accounted for by stratifying patients across samples according to number of contributing items and/or the use of a non-parametric Kruskal-Wallis test.

## Conclusions

TDC allows a clinician to decide on the end point of treatment in a way that attaining functional status by a sufficiently large relative improvement in score levels will be concomitant with a perception of a substantial treatment effect by chronic pain patients. In combination with evaluation by a blinded assessor, TDC enables randomized controlled trials with therapies that have a variable therapy- and patient-specific duration.

## Appendix

### Averaging of ratio values between scores from two times of measurement

Three possible methods of averaging ratio values between subsequent score values are: (1) calculating the ratio between the mean score values at the second time of measurement and the mean baseline scores, (2) determining the mean of the ratio values of the pairs of successive score values from various items, and (3) determining the mean of the ratio values under (2) that have first been transformed to attain a zero point to which values of an equivalent relative increase and decrease have the same distance.

In order to illustrate the three methods of averaging, Table 6 shows numerical examples of three items at two times, i.e. scores *S*_*1,i*_ and *S*_*2,i*_ (in which *i* is the item number, *i = 1 .. 3*), with three values of the ratio between *S*_*2,i*_ and *S*_*1,i*_ which correspond with three multiplication factors to describe the transition from *S*_*1,i*_ to *S*_*2,i*_. The multiplication factors chosen are 2 (increase by a factor of 2), 1 (no change) or 0.5 (decrease by a factor of 2). Three situations are shown: (i) equal values of the three items at baseline (*S*_*1,*i_-values, Table 6, top), (ii) a smaller, intermediate and a larger *S*_*1,i*_-value which are subjected to multiplication by 2, 1 and 0.5 respectively (middle), or (iii) to multiplication by 0.5, 1 and 2 respectively (bottom). Table 6 also gives the mean *S*_*1,i*_- and *S*_*2,i*_-scores and the ratio between both mean values (Method (1)), and the mean of the multiplication factors (mean of *Ri* values, Method (2)). Furthermore, Table 6 shows logarithmical transformed values of the multiplication factors (*log(Ri)*) and the mean of these transformed values (Method (3)). The multiplication factors have also been transformed to Contrast-values (*C*_*i*_ *= (R*_*i*_*- 1)/(R*_*i*_ *+ 1)*), and the mean of the Contrast-values is shown (second variant of Method (3)).

**Table 6:** Three possible methods of averaging ratios between successive score values

***equal baseline scores:***					
	*S*_*1,i*_	ratio (*R*_*i*_)	*S*_*2,i*_	*log(R*_*i*_*)*	*C*_*i*_ *= (R*_*i*_*-1)/(R*_*i*_ *+ 1)*
	50	2	100	0.301	0.333
	50	1	50	0.000	0.000
	50	0.5	25	0.301	-0.333
*Method (1):*	mean *S*_*1,i*_ = 50.0				*Method (2):* mean of *R*_*i*_ values: 1.17
	mean *S*_*2,i*_ = 58.3				
	(mean *S*_*2,i*_)/(mean *S*_*1,i*_) = 1.17				
*Method (3):*	mean of *log(R*_*i*_*)* values: 0.000 ; mean *R*: 1.00				
			(re-transformed mean log-value:10^(mean log(Ri)^)		
	mean of *C*_*i*_ values: 0.000 ; mean *R*: 1.00				
			(re-transformed mean *C*-value: *(1 + C)/(1-C)*)		
***non-equal baseline scores, largest baseline with smallest R***_***i***_ ***value:***					
	*S*_*1,i*_	ratio (*R*_*i*_)	*S*_*2,i*_	*log(R*_*i*_*)*	*C*_*i*_ *= (R*_*i*_*-1)/(R*_*i*_ *+ 1)*
	20	2	40	0.301	0.333
	40	1	40	0.000	0.000
	60	0.5	30	-0.301	-0.333
*Method (1):*	mean *S*_*1,i*_ = 40.0				*Method (2):* mean of *R*_*i*_ values: 1.17
	mean *S*_*2,i*_ = 36.7				
	(mean *S*_*2,i*_)/(mean *S*_*1,i*_) = 0.917				
*Method (3):*	mean of *log(R*_*i*_*)* values: 0.000 ; mean *R*: 1.00				
			(re-transformed mean log-value:10^(mean log(Ri)^)		
	mean of *C*_*i*_ values: 0.000 ; mean *R*: 1.00				
			(re-transformed mean *C*-value: *(1 + C)/(1-C)*)		
***non-equal baseline scores, largest baseline with largest R***_***i***_ ***value:***					
	*S*_*1,i*_	ratio (*R*_*i*_)	*S*_*2,i*_	*log(R*_*i*_*)*	*C*_*i*_ *= (R*_*i*_*-1)/(R*_*i*_ *+ 1)*
	20	0.5	10	0.301	0.333
	40	1	40	0.000	0.000
	60	2	120	-0.301	-0.333
*Method (1):*	mean *S*_*1,i*_ = 40.0				*Method (2):* mean of *R*_*i*_ values: 1.17
	mean *S*_*2,i*_ = 56.7				
	(mean *S*_*2,i*_)/(mean *S*_*1,i*_) = 1.42				
*Method (3):*	mean of *log(R*_*i*_*)* values: 0.000 ; mean *R*: 1.00				
			(re-transformed mean log-value:10^(mean log(Ri)^)		
	mean of *C*_*i*_ values: 0.000 ; mean *R*: 1.00				
			(re-transformed mean *C*-value: *(1 + C)/(1-C)*)		

*S*_*1,i*_ and *S*_*2,i*_, subsequent score values of three items ( *i = 1..3*) at times ‘1’ and ‘2’ respectively. *R*_*i*_, ratio between *S*_*2,i*_ and *S*_*1,i*_ (multiplication factor of *S*_*1,i*_). *log(R*_*i*_*)*, logarithmically transformed *R*_*i*_-values. *C*_*i*_, *R*_*i*_-values transformed as Contrast-values. Three methods of averaging ratio values are illlustrated; Methods (3) includes two variants of data transformation, logarithmically and as Contrast. For further explanation, see text.

When all values of *S*_*1,i*_ are equal (Table 6, top), the ratio between the mean *S*_*2,i*_-value and the mean *S*_*1,i*_-value (Method (1)) equals the mean of the multiplication values, *Ri* (Method (2)). When the values of *S*_*1,i*_ differ, the outcome of the ratio between the mean *S*_*2,i*_ values and the mean *S*_*1,i*_ values largely depends on how the largest *S*_*1,i*_ value is modulated. If this value is multiplied by a factor 0.5 (a decrease, Table 6, middle), this ratio is smaller than the mean of the multiplication factors. Reversely, if the largest *S*_*1,i*_ value is multiplied by the factor 2 (an increase, Table 6, bottom), the ratio between the mean *S*_*2,i*_-values and the mean *S*_*1,i*_-values is larger than the mean of the multiplication factors. The mean value of the logarithmically transformed factor values is zero because the transformed values of an equivalent decrease and increase by a factor of 2 have the same distance on both sides of the zero point of log-values. The multiplication factor 1 which reflects no change becomes also zero as a log-value. When multiplication factors are transformed to Contrast-values, there is, like for log-values, also a zero point to which an equivalent relative increase and decrease have the same distance. Thus the mean of the transformed factors underlying these changes is zero.

Thus Method (1) of averaging ratios (determining the ratio between mean *S*_*2,i*_ and mean *S*_*1,i*_) is dominated by the change in the score which has the largest baseline value. Method (1) is also arbitrary as weighing factors are unknown by which scores from different items might be summed and subsequently averaged adequately. The second and the third method are invariant to the baseline levels of the scores as only the ratio values from the various pairs of successive scores are considered. However, the second method (mean of ratio values) might yield an inappropriate outcome regarding the overall tendency of the multiplication factors. For example, when the score of a first item shows an increase by a multiplication factor of 2 and a second item shows a decrease by a factor of 2 (multiplication factor 0.5), the mean of the values 2 and 0.5 equals 1.25. This outcome indicates an overall factor of relative increase for the various pairs of subsequent scores as a mean of 1.25 is larger than a mean factor of 1 that represents no relative change. The reason for this inadequate outcome is that the values of 2 and 0.5, corresponding with an equivalent relative increase and decrease in score value, do not have the same distance to the zero point of ratio values. When the ratio values 2 and 0.5 are first transformed (method (3)), for example logarithmically, the mean of the log-values of 2 and 0.5 (+0.303 and −0.303) becomes zero. In general, when a score increases by a factor *k* while a score from another item decreases by the same factor *k* (multiplication by *1/k*), the logarithmic values of the multiplication factors *k* and 1/*k* are equidistant with respect to the zero log-value as *log(k*) = −*log(1/k*).

Thus ratio values have been transformed in the present study to attain an index of averaged transformed multiplication factors of which the value zero reflects an overall factor related to no change. When the mean log-value which is zero in the abovementioned examples, is transformed back, the value of the overall multiplication factor is 1, representing no relative change indeed. A positive mean of transformed factor-values and a negative mean value are related to an overall relative increase and decrease respectively in the ratios (multiplication factors) between various pairs of subsequent score levels, regardless of the score levels themselves. A positive mean of transformed score values corresponds to an increase in pain or impairment, whereas a negative mean corresponds to a decrease.

However, the logarithmic ratio value from two successive scores within a patient (*log(R*_*i*_*) = log(S*_*2,i*_*/ S*_*1,i*_*)*_,_ in which *S*_*2,i*_ is the second score of item *i,* and *R*_*i*_ the ratio between both scores) will be undefined (infinite negative) when *S*_*2,i*_ becomes zero after a complete disappearance of pain or impairment. In order to avoid undefined values, a Contrast (*C*) value between inter-visit scores was determined for each item that contributed to the average. The value of *C*_*i*_, a normalized difference value between two measurements of item *i*, was given by:

$$C_{i}=\left(S_{2,i}-S_{1,i}\right)/\left(S_{2,i}+S_{1,i}\right)\;,$$

in which *S*_*1,i*_ is the reference score of the *i*-th item and *S*_*2,i*_ the score at a later visit.

The possible values of C_*i*_ vary within a range from −1 to +1. C_*i*_ is related to the relative change in score values. Equation (1) can be rewritten as C_*i*_ = (R_*i*_ - 1)/(R_*i*_ + 1) in which R_*i*_ is the ratio between S_2,*i*_ and S_1,*i*_. Like for logarithmically transformed values, an increase of a score by a factor *k* is equivalent to a decrease by this factor *k* when scores are transformed to *C*-values as, apart from the sign, both *C*-values are equidistant to the zero-point. (see Table 6 for a numerical example with a factor of 2). Thus with an increase of the reference score by a factor *k*, *C = (k - 1)/(k + 1)*, according to equation (1), and with a decrease by a factor *k* (multiplication by *1/k*), *C’ = (1-k)/(1 + k) = −C*.

**Figure 6:**
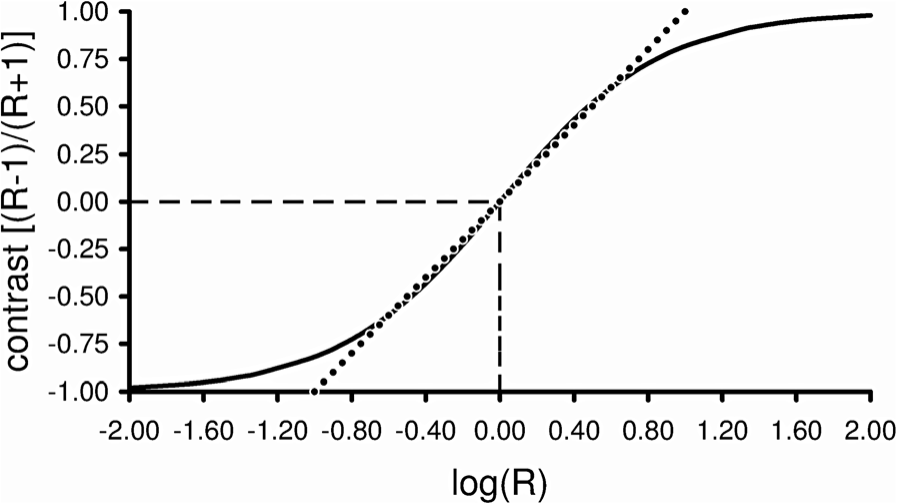
**Contrast-value as a function of logarithmic value of the ratio between two score values (solid curve).** Ratio R = S_2_/S_1_, in which S_1_ and S_2_ are the first and the second score value. When there is no difference between S_2_ and S_1_, both the Contrast-value and the value of log(R) are zero (hatched lines). When S_2_ is zero and S_1_ has a positive value, log(R) is undefined (infinite negative) whereas the Contrast-value is −1.0. When S_2_ has a positive value while S_1_ is zero, log(R) becomes undefined (infinite positive) whereas the Contrast-value is +1.0. If all Contrast-values were numerically identical to log(R) values, the relationship between Contrast and log(R) values would be depicted by he dotted straight line. Note that although not identical in general, Contrast and log(R) values are numerically similar (deviation with respect to the mean of both types of values < 15%) for a wide range of log(R) values on both sides of the zero point (range −0.80 to +0.80).

C_*i*_ values and logarithmic ratio values between *S*_*2,i*_ and *S*_*1,i*_ are numerically similar within a wide range of logarithmic ratio values on both sides of the zero point (Figure 6). As a numerical example of C_*i*_: before treatment, a patient scored a pain level of ‘4’ after palpation of the left superficial masseter muscle. Since the score became ‘2’ at a later visit, C_*i*_ of this item was −0.333 (= (2–4)/2 + 4) = −2/6). *Ci*, in this example −0.333, is similar to the logarithmic value of the ratio between both scores, log(2/4) = −0.301.

At the various treatment visits, actual score values from a later visit were compared with respect to the reference values. Only those items were considered that were related to sufficiently pronounced signs or symptoms (reference items).

For each reference item *i*, the Contrast-value C_*i*_ was determined according to equation (1). For each treatment or follow-up visit of a patient, all C_*i*_ values were averaged to obtain a single summarizing index, ‘Treatment Duration Control’ (TDC):

$$\mathrm{TDC}=\left(\sum_{i=1}^{n}C_{i}\right)/n,$$

in which *n* is the number of items.

TDC is related to an overall ratio (multiplication factor) between various pairs of subsequent score levels from the reference items. Thus TDC is invariant to the score levels themselves. The name of the index ‘Treatment Duration Control’ refers to the use of cut-off values of TDC for controlling treatment duration.

For Contrast-values which are sufficiently remote from the extreme values of −1 or +1 (−0.8 < *C*_*i*_ < 0.8), each term *C*_*i*_ in equation (2) is approximated well by *log(R*_*i*_*)* (Figure 6). TDC can then be approximated by:

$$\mathrm{TDC}\approx \left(\sum_{i=1}^{n}\log\left(R_{i}\right)\right)/n,$$

thus TDC is then approximately the logarithmic value of the geometric mean of the ratios *R*_*i*_ (*i = 1…n*; geometric mean: $\mathrm{GM}={\left(\prod_{i=1}^{n}\mathrm{Ri}\right)}^{1/n}$) between the various pairs of subsequent score values. The geometric mean of the ratios (the global multiplication factor related to TDC) corresponds approximately to the median of the ratios if the log transformed ratios have a (roughly) symmetrical distribution.

One might argue that percentage change might be used as a measure of relative change rather than Contrast. A percentage increase is, however, apart from the sign not equivalent to the same percentage decrease in terms of multiplication factors. As a numerical example, 20% increase of a score S_1_ of 100 arbitrary units means that S_2_ will be 120 units. Thus the transition of S_1_ to S_2_ can be described by multiplying S_1_ by a factor of 1.2 (120/100). A decrease of 20% means that S_1_ of 100 units becomes S_2_ of 80 units. This transition can be described by S_1_ multiplied by a factor of 0.8. The reversed value of this factor equals 1.25 (1/0.8) which is unequal to the multiplication factor of 1.2 describing an increase of 20%. A transformation of ratio values between successive score-values to Contrast-values remains most appropriate to average values of relative change across various reference items.

### Lack of bias by regression to the mean in Contrast and TDC-values

In order to avoid random fluctuations in TDC-values, items with a sufficiently large starting value *S*_*1,i*_ have been selected as ‘reference items’. One might argue that such a selection might introduce the risk on regression to the mean and might cause bias in the treatment outcome. Such a mechanism by chance is, in part, involved in a decrease in a raw score value following treatment, which has initially a large value (by merely having a large initial value such a score has a larger chance to decrease than to increase, irrespective of any treatment effect). A significant linear regression with a negative gradient occurred in the relationship between the raw differences in a later score and the baseline one and the baseline scores from the various patients. For example, the difference in VAS score of predominant pain between the last measurement following treatment (score *S*_*2*_) and baseline (score *S*_*1*_) decreased on average according to the regression function: (*S*_*2*_ - *S*_*1*_) = −0.709.*S*_*1*_ + 4.337, with a Pearson’s correlation coefficient of 0.601 (n = 118). In combination with a possible treatment effect, this significant (p < 0.001) regression is thus, in part, due to regression to the mean by chance.

Ignoring the scatter in the post-treatment scores (*S*_*2*_) for the moment, a relationship between difference in scores and the corresponding baseline score (S_*1*_) can be described by:

(3) $$\left(\bar{S_{2}-S_{1}}\right)=a.S_{1}+b,$$

in which *a* is the gradient, *b* the intercept, and $\left(\bar{S_{2}-S_{1}}\right)$ is the mean difference between *S*_*2*_ and S_*1*_ that occurs with S_*1*_ according to the regression function. In this mean difference, *S*_*1*_ is a value from an individual patient whereas the corresponding value of *S*_*2*_ is a mean value which is further denoted as $\bar{S_{2}}$.

From equation (3) it follows that the ratio between $\bar{S_{2}}$ and *S*_*1*_ is given by:

(4) $$\bar{S_{2}}/S_{1}=\left(a+1\right)+b=c,$$

in which *c* is a constant. Thus the ratio between the mean value $\bar{S_{2}}$ and the corresponding baseline score *S*_*1*_ is constant, and this ratio does not depend on the corresponding value of *S*_*1*_. The logarithmic value of the ratio is also constant:

(5) $$\log\left(\bar{S_{2}}/S_{1}\right)=\log\left(c\right)=c’$$

Contrast-values between $\bar{S_{2}}$ and *S*_*1*_ which are sufficiently remote from the extreme values of −1 or +1 (−0.8 < C_*i*_ < 0.8), approximates the logarithmic ratio value (see Appendix section above), hence:

(6) $$\left(\bar{S_{2}}-S_{1}\right)/\left(\bar{S_{2}}+1\right)\approx \log\left(\bar{S_{2}}\right)/S_{1}=c’$$

Thus the Contrast-values are also independent from their corresponding values of *S*_*1*_.

In the abovementioned relationship between pre- and post-treatment VAS scores of predominant pain intensity on a 100 mm scale, scatter is involved in the *S*_*2*_-values. While the relationship between the difference in score values (*S*_*2*_ - *S*_*1*_) and the corresponding baseline score (*S*_*1*_) yielded the regression function:

(*S*_*2*_ - *S*_*1*_) = −0.709.*S*_*1*_ + 4.337 (r = 0.601, n = 118, p < 0.001), the relationship between the ratio *S*_*2*_/ *S*_*1*_ and *S*_*1*_ of the VAS-scores was described by the regression function:

*S*_*2*_/ *S*_*1*_ = −0.00592. *S*_*1*_ + 0.709 (r = 0.171, n = 118, not significant). The gradient of this relationship was less steep than the gradient of the relationship between (*S*_*2*_ - *S*_*1*_) and *S*_*1*_ (equation (3)), and the regression was non-significant, which could be expected according to equation (4). However, the scatter of the ratio values (*S*_*2*_/*S*_*1*_) became smaller the larger the *S*_*1*_ values, and was large for small *S*_*1*_ values in particular.

The relationship between the Contrast-values [(*S*_*2*_ - *S*_*1*_)/(*S*_*2*_ + *S*_*1*_)] and *S*_*1*_ was described by the regression function:

(*S*_*2*_ - *S*_*1*_)/(*S*_*2*_ + *S*_*1*_) = −0.000829.S1 – 0.540 (r = 0.0387, n = 118, not significant). The regression line of this relationship was nearly horizontal. Furthermore, the band of scatter of the Contrast-values was similar within the entire range of *S*_*1*_ values, because (in contrast to ratio values) the distribution of Contrast-values is symmetrical. Furthermore, Contrast-values are limited between −1 and +1, thus large deviations from the regression line were avoided. The constant bandwidth of scatter of the Contrast-values around the nearly horizontal regression line (which approximates the mean of the Contrast-values) means that, following treatment, a similar variety of relative change in pain intensity occurs in different patients, regardless of the baseline level of pain intensity. Thus by transforming difference values to Contrast-values (normalized difference values) any regression, also one to the mean which occurred in the difference values as a function of the baseline values, is eliminated.

A regression in the relationship between Contrast-value and baseline value of the item will be lacking for any item according to equation (6), with a constant bandwidth of scatter because of the use of Contrast-values. Thus the index TDC being the mean of Contrast-values from several items (equation (2)) includes components that are all invariant to each of their baseline values. For each patient only those items were included in TDC that were related to sufficiently pronounced signs or symptoms of that patient (reference items). The baseline of each of these selected items is therefore related to the patient’s baseline of severity of myogenous TMD, and all the patient’s Contrast-values are invariant to the baselines of the corresponding items as well as to this baseline severity. A regression will therefore be lacking in the relationship between TDC and the baseline of an item which can be used to characterize the baseline severity of myogenous TMD of the various patients in the sample. Such characterizing items are the predominant pain of the masticatory system (VAS-scores) and to some extent generic Health-related Quality of Life (EQ-5D utility units). A lack of regression has been observed indeed, between TDC and pain intensity and HRQoL respectively (Figures 4A-B). The constant bandwidth of scatter of post-treatment TDC-values around the nearly horizontal regression line means that, a similar variety of TDC-values from different patients occurs, regardless of the severity level of myogenous TMD. Patients whose TDC-value has dropped to or beyond the cut-off level of −0.379, only have attained functional status. With a constant bandwidth of scatter, the fraction of patient whose TDC ≤ −0.379 is constant and the chance of attaining functional status is therefore independent from the baseline severity level of myogenous TMD of the patient.

### Scores from anamnesis and clinical examination

The anamnestic questionnaire included scoring on adjectival 0–4 point scales (Table 1) of frequency of pain from the masticatory system, stiffness or fatigue of the jaw muscles and limitations to movement of the jaw. Furthermore, the extent of impairment of chewing hard food and yawning respectively was scored. The questionnaire also included scoring of the intensity of the predominant pain from the masticatory system on a 100 mm Visual Analogue Scale (VAS; anchor points: ‘no pain’ and ‘the most intense pain one can imagine’). The total number of anamnestic items related to myogenous TMD, was 6 of which 5 were scored on 0–4 point scales.

The clinical examination included scoring of pain intensity on a adjectival 0–4 point scale during: (i) active and passive jaw movements in vertical, lateral and anterior-posterior directions, (ii) palpation of the deep and superficial masseter muscles, the anterior and posterior temporalis muscles, the sternocleidomastoid and the attachment of various muscles on the occipital bone, and (iii) after the patient had been instructed to clench in eccentric positions as well as in maximal occlusion. As pain was scored for both sides and palpation and most jaw movements were also side-related, the total number of clinical items was 42.

### Choosing two cut-off points of TDC

Two cut-off points of TDC are necessary to control treatment duration. A first cut-off point was used to decide whether a patient had responded sufficiently following a treatment-specific time interval. A second cut-off point served to decide when the upper limit of functional status had passed and the treatment had become potentially ‘successful’.

Regarding the first cut-off point of TDC: for a treatment which has a moderate effect at most, the majority of the end scores will be ‘2’ or ‘3’ in patients where an initial level of ‘3’ or ‘4’ predominated on an adjectival 0–4 scale of extent or frequency of pain or impaired function. The end situation corresponds to a patient’s condition in which ‘moderate/regularly’ pain and/or impaired function occurs. TDC = 0.212 was chosen as the first cut-off point to describe approximately the mean of Contrast-values of the transitions in score values from ‘4’ to ‘3’ (Contrast: -0.143 = (3 – 4)/(3 + 4)), ‘4’ to ‘2’ (C: -0.333), and ‘3’ to ‘2’ (C: -0.200). The cut-off point TDC = −0.212 corresponds to a decrease of 35% in a single score of pain intensity at a 100 mm VAS [−0.212 = (65 – 100)/(65 + 100)]. If a patient’s TDC was larger than −0.212 (TDC > −0.212) at a critical stage of treatment, the patient was insufficiently responsive (a less negative value than −0.212 means less change towards recovery).

Regarding the second cut-off point of TDC, the upper limit of functional status (*ULFS*) must first be determined. Functional status in myogenous Temporomandibular Disorders is characterized by a low level of signs and symptoms that might occasionally occur in healthy subjects. Normative data of the items from Community Control (CoCo) subjects were lacking Thus *ULFS* could not be assessed by determining the 95th percentile of score values from CoCos following the same anamnestic and clinical examination as used for the TMD patients. However *ULFS* could be approximated in the following way. In the adjectival 0–4 point scales used in the present study, the score ‘0’ means ‘no pain or impairment’ and the score ‘1’ means ‘slight’ pain or ‘sometimes’ painful or ‘sometimes’ impairment and the score ‘2’ means ‘moderate’ pain, ‘regularly’ painful or ‘regularly’ impairment (Table 1). A panel of five dentists specialized in TMD agreed that a functional status is characterized by a large majority of scores with end values of less than 2 units. After considering the post-treatment score values of 20 patients from a pilot sample, a mean score level of 1.40 units was assessed for *ULFS*.

The following procedure was followed to select a patient from the pilot sample, with overall maximal signs and symptoms of myogenous TMD, and to determine the cut-off value of TDC related to the factor *T* required to attain *ULFS* from baseline. Score values from 0–4 point scales were available for 5 anamnestic items and 42 clinical items. Items with sufficiently large score values at baseline (i.e. a score value of 2, 3 or 4, which exceeded the smallest detectable difference (SDD; see the main text for more details) were selected for each patient as reference items for calculating Contrasts and the TDC-value. The patients could be ranked according to their overall level of signs and symptoms by considering the summed score value of the reference items at baseline as a primary ranking criterion and the number of reference items as a secondary criterion. The patient with maximal signs and symptoms had a summed score value of 104 to which 32 items contributed, i.e. 7 scores with a value ‘2’ , 10 scores of ‘3’ , and 15 scores of ‘4’ . The Contrast between a score value of 2 and the level of *ULFS* (1.40 units) is −0.176 (= (1.4 - 2)/(1.4 + 2)), and the Contrast-values are −0.364 and −0.481 for a baseline score value of ‘3’ and ‘4’ respectively. The mean of all Contrast-values of the patient with the abovementioned score profile yields the TDC-value of −0.378 (= [−7 × 0.176 –10 × 0.364 – 15 × 0.481]/32). This TDC-value corresponds with a value for *1/T* of 0.451 (= [1 + (−0.378)]/[1 - (−0.378)]; hence a treatment factor *T* of 2.22) and to a decrease of 54.9% in a single score value of pain intensity (= (1–0.451)*100%). The value of the decrease in pain intensity has been rounded off to 55% and hence the value −0.379 was chosen as a cut-off point of TDC for deciding whether *ULFS* had been passed during treatment of the patients from the present study.

In retrospect, two patients from the large sample of 118 patients from the present study, had overall a higher level of signs and symptoms (score profiles of baseline reference items: 1 × 2, 8 × 3, 30 × 4, and 7 × 2, 9 × 3, 17 × 4) than the patient from the pilot sample (7 × 2, 10 × 3, 15 × 4). Their sum value was 146 and 109 and the number of reference items was 39 and 33 respectively rather than 104 (sum value) and 32 (number of reference items) of the patient from the pilot sample. However, their mean score level across the reference items (94% and 83%; 100% means a maximal score value of 4 for all items) was not increased much with respect to the mean of the patient from the pilot sample (81%), because many items had a maximal score value of 4 units. The cut-off values of TDC with respect to an *ULFS* value of 1.40 were therefore only slightly more negative for the two patients from the current sample than for the patient from the pilot sample, i.e. TDC = −0.449 (62.0% decrease in a single score of pain intensity), TDC = −0.385 (56.1% decrease) rather than TDC = −0.378 (54.9% decrease).

Furthermore, distributions of post-treatment scores (from the last follow-up visit) pooled across patients from the present study whose treatment was successful in the long-term, provided information on the Upper Limit of Functional Status (*ULFS*) in retrospect.

First, the post-treatment scores of only the reference items were considered for each patient, in total 786 scores from 66 patients. These scores had initially a high score level of 2, 3 or 4 units on a 0–4 point scale, reflecting pronounced signs and symptoms of the patients, and, had in general a low level following a successful treatment. The frequency of reference items from the various patients of the entire sample (patients with and without a successful treatment) differed between items, i.e. it varied within a range from 47% to 82% for items related to anamnestic questions and from 6% to 69% for items related to clinical examination. In order to account for these differences between items in the chance of being a reference item in myogenous TMD patients, the number of any post-treatment value of a reference item was weighted according to this chance. The 95th percentile of the weighted distribution of post-treatment scores of reference items from the patients with a successful treatment, was 1.66 units. This upper limit of 1.66 units of residual score values is only slightly larger than the value of 1.40 units which was *a priori* attributed to *ULFS* by the panel, based on post-treatment scores from the pilot sample of 20 patients (see above). The current TDC-procedure based on an *ULFS* of 1.40 units was so effective that this level confined 92.5% of the post-treatment scores of reference items in patients whose treatment was successful according to the criterion TDC ≤ −0.379.

Second, all post-treatment scores were considered which were pooled across the patients whose treatment was successful (in total 3102 scores from 66 patients). Thus apart from reference scores, also scores which had initially small values of 0 or 1 units were considered. In order to diminish the influence of post-treatment score values from items which were rarely involved as reference items (thus attenuating the possible influence of many zero values from those items which rarely detect signs and symptoms), the number of any post-treatment value of any item was weighted again according to the chance of this item of being a reference item. The 95th percentile of the weighted distribution of post-treatment scores of all items was 1.08 units. As this distribution might approach the one from Community Controls, this finding suggests that the actual value of *ULFS* might be somewhat smaller than 1.40 units for myogenous TMD. A smaller value of *ULFS* means a more negative cut-off value for TDC for enabling the score level of a patient with maximal signs and symptoms to attain *ULFS* following treatment. Using the pre-treatment scores of the patient with maximal signs and symptoms in the pilot sample, the cut-off value of TDC is −0.486 (65.5% decrease in a single score of pain intensity) with a value of 1.08 units for *ULFS*, rather than −0.378 (54.9% decrease) with an *ULFS* value of 1.40 units.

The conclusion of a more negative cut-off for TDC is reinforced by another finding from the present study, i.e. that the post-treatment distribution of the TDC-values became bimodal in the long-term, representing two groups of patients in respect of a successful/unsuccessful treatment (Figures 3B, C and D). The finding of a slightly more negative TDC-values of −0.420 to −0.560 at the trough of the distribution (corresponding to 59% and 72% decrease respectively in a single score of pain intensity) than TDC = −0.379 (decrease 55%) suggests that the separation between the two groups might have been better still, had a slightly more negative cut-off point been used.

However, the criterion of TDC ≤ −0.379 has been used in combination with a discrepancy rule in which occasionally the patient’s demand for subsequent treatment overruled the conclusions of the clinical examination. The patient’s opinion as reflected in anamnestic items on daily functioning of the oral system was given priority in treatment outcome if the index of overall relative change (including changes related to items from clinical tests) indicated a ‘successful’ treatment while the anamnestic items alone indicated an ‘unsuccessful’ treatment (for details, see the main text). The use of a slightly less conservative criterion TDC ≤ −0.379 in combination with the discrepancy rule was actually more effective than a more negative TDC-value as cut-off. Apart from a few patients who profoundly expressed a demand for subsequent treatment in their scores of anamnestic items and could overrule the conclusions of the clinical examination, over-treatment of a few other patients was prevented (cf. Discussion).

### Inclusion and exclusion criteria of the patients

The patients with myogenous TMD, a chronic pain disorder, met the following inclusion and exclusion criteria (cf. ref. [17]): (i) pain and tenderness of the muscles of mastication and restricted mandibular opening of 3 month duration or longer, (ii) no clinical and/or radiographic evidence of organic TMJ changes, (iii) no previous TMD treatment or recent (< 1 year) other pain treatment, (iv) no evidence of serious psychopathology (no psychotherapy and/or psycho-medication, no recent dramatic life events), and (v) between 18 and 65 years of age. The mean age of the patients was 31.6 years (SD 10.0); 93% were female. The median duration of pre-treatment pain was 1.1 years (range 3 months to 20 years).

### The use of TDC on scales with a reversed meaning

In the present study, Contrast-values have been determined for scores of items that have always a zero value when there is no pain or impairment and a maximal value when the extent of pain or impairment would be the greatest. Improvement corresponds therefore always with a decrease in score value. However, scores from other scales than used in the present study might have a reversed meaning. For example, the better an aspect of quality of life, the larger a score value will be in Euroqol [35]. In order to have a consistent meaning of lacking any impairment for a Contrast-value that equals −1, a score value related to quality of life could be transformed to a complementary value by calculating the difference between the maximum value of the scale and the actual score value. Thus, the transformed values for the first and the second score value are given by S_1,*i*_’ = S_max_-S_1,*i*_ and S_2,*i*_’ = S_max_-S_2,*i*_ respectively, and the Contrast-value (C_*i*_) of item *i* is given by C_*i*_ = (S_2,*i*_’-S_1,*i*_’)/(S_2,*i*_’ + S_1,*i*_’).

For example, in Euroqol [35], the self-perceived health status is recorded on a vertical Visual Analogue Scale of 100 units with an anchor point at the bottom of “worst imaginable status of health” and with an anchor point at the top of “best imaginable status of health” (EQ-5D_vas_). Suppose that the patient’s score values are 40 units (S_1,*i*_) before and 80 units after treatment (S_2,*i*_). With a maximum score value of 100 units, the complimentary values related to ‘extent of impairment of status of health’ are then S_1,*i*_’ = 60 (= 100 – 40) and S_2,*I*_ ’ = 20 (= 100 – 80). The Contrast-value of the complementary scores (C_*i*_) is −0.500 (= (20 – 60)/(20 + 60)).

### The use of TDC when *a priori* knowledge of an item’s unimpaired value is lacking

The question is how to determine a Contrast-value when the value of a score corresponding to ‘least impairment’ is unknown. For example, due to pathology of the Temporomandibular joint, a patient of the type of artrogenous TMD might have a restricted ability to open the mouth maximally. Apart from pain variables, the extent of maximal mouth opening is then a key factor to include in TDC for controlling treatment duration. However, the unrestricted maximal mouth opening is *a priori* not known for a particular patient. An approximate Contrast-value might then be determined by taking, at least initially, a lower 95% confidence limit (CL) of normal values (determined in a group of healthy subjects) as score value for unrestricted maximal mouth opening, thus S_CL-normal_ is initially taken as the maximum score value of the scale, S_max_. Because, like score values from a quality of life scale (see above, Appendix section ‘The use of TDC on scales with a reversed meaning’), a larger score value of mouth opening is related to less impairment, the first and the second score are again transformed to complementary values according to: S_1,*i*_’ = S_max_ - S_1,*i*_ and S_2,*i*_’ = S_max_ - S_2,*i*_, in which S_max_ = S_CL-normal_. The Contrast-value C_*i*_ for maximal mouth opening is then given by C_*i*_ = (S_2,*i*_’ - S_1,*i*_’)/(S_2,*i*_’ + S_1,*i*_’). When a patient’s value of S_2,*i*_ would become larger than S_CL-normal_ in a late phase of treatment, S_max_ in the transformation equation might then be replaced by this S_2,*i*_ value for improving the estimated Contrast-value for the actual visit and subsequent visits.

For example, suppose that the patient’s score values for mouth opening are 20 mm (S_1,*i*_) before and 30 mm halfway treatment (S_2,*i*_). With a lower 95% confidence limit for normal values of 35 mm [36], initially taken as S_max_, the complementary values related to ‘extent of impairment of mouth opening’ are then S_1,*i*_’ = 15 (= 35–20) and S_2,*i*_’ = 5 (= 35–30). The Contrast-value of the complementary scores (C_*i*_) is −0.500 (= (5–15)/(5 + 15)). Suppose that S_2,*i*_ becomes 43 mm at the end of treatment reflecting that the patient’s value has entered the 95% range of normal values (between 35 and 45 mm). This novel value of S_2,*i*_ is then taken as S_max_, the complementary values are then S_1,*i*_’ = 23 (= 43–20) and S_2,*i*_’ = 0 (= 43–43), and C_*i*_ will be −1 (= (0–23)/ (0 + 23)) indicating a lack of any impairment with respect to the ability of opening the mouth maximally.

## Competing interests

Both authors declare that they have no competing interests.

## Authors’ contributions

HG conceived the concept of the index ‘Treatment Duration Control’ and the related RCT procedure, contributed to the data analysis and prepared the manuscript. RG was the ‘investigator’ in the RCT, collected the data, performed data analysis, helped interpreting the results and preparing the manuscript. Both authors have read and approved the final version of the manuscript.

## Pre-publication history

The pre-publication history for this paper can be accessed here:

http://www.biomedcentral.com/1471-2288/13/123/prepub
